# An Integrated AI Framework for Occupational Health: Predicting Burnout, Long COVID, and Extended Sick Leave in Healthcare Workers

**DOI:** 10.3390/healthcare13182266

**Published:** 2025-09-10

**Authors:** Maria Valentina Popa, Călin Gheorghe Buzea, Irina Luciana Gurzu, Camer Salim, Bogdan Gurzu, Dragoș Ioan Rusu, Lăcrămioara Ochiuz, Letiția Doina Duceac

**Affiliations:** 1 Doctoral School of Biomedical Sciences, “Dunărea de Jos” University of Galați, 800008 Galați, Romania; maria_valentina_popa@yahoo.com; 2 National Institute of Research and Development for Technical Physics, IFT Iași, 700050 Iași, Romania; calinb2003@yahoo.com; 3 Clinical Emergency Hospital “Prof. Dr. Nicolae Oblu” Iași, 700309 Iași, Romania; 4 Department of Preventive Medicine and Interdisciplinarity, Discipline of Occupational Health, “Grigore T. Popa” University of Medicine and Pharmacy, 700115 Iasi, Romania; 5 Faculty of Medicine, “Ovidius” University of Constanța, 900601 Constanța, Romania; 6 Department of Morphofunctional Sciences, Faculty of Medicine, “Grigore T. Popa” University of Medicine and Pharmacy, 700115 Iasi, Romania; bgurzu@yahoo.com; 7 Department of Environmental Engineering, Mechanical Engineering and Agritourism, Faculty of Engineering, “Vasile Alecsandri” University of Bacău, 600115 Bacău, Romania; drusu@ub.ro; 8 Faculty of Medicine and Pharmacy, “Grigore T. Popa” University of Medicine and Pharmacy Iași, 700115 Iași, Romania; lacramioara.ochiuz@umfiasi.ro; 9 Faculty of Medicine and Pharmacy, “Dunărea de Jos” University of Galați, 800008 Galați, Romania; letimedr@yahoo.com

**Keywords:** occupational health risk prediction, burnout and Long COVID in healthcare workers, tabular deep-learning models, explainable artificial intelligence (XAI), workforce planning and decision support

## Abstract

Background: Healthcare workers face multiple, interlinked occupational health risks—burnout, post-COVID-19 sequelae (Long COVID), and extended medical leave. These outcomes often share predictors, contribute to each other, and, together, impact workforce capacity. Yet, existing tools typically address them in isolation. Objective: The objective of this study to develop and deploy an integrated, explainable artificial intelligence (AI) framework that predicts these three outcomes using the same structured occupational health dataset, enabling unified workforce risk monitoring. Methods: We analyzed data from 1244 Romanian healthcare professionals with 14 demographic, occupational, lifestyle, and comorbidity features. For each outcome, we trained a separate predictive model within a common framework: (1) a lightweight transformer neural network with hyperparameter optimization, (2) a transformer with multi-head attention, and (3) a stacked ensemble combining transformer, XGBoost, and logistic regression. The data were SMOTE-balanced and evaluated on held-out test sets using Accuracy, ROC-AUC, and F1-score, with 10,000-iteration bootstrap testing for statistical significance. Results: The stacked ensemble achieved the highest performance: ROC AUC = 0.70 (burnout), 0.93 (Long COVID), and 0.93 (extended leave). The F1 scores were >0.89 for Long COVID and extended leave, whereas the performance gains for burnout were comparatively modest, reflecting the multidimensional and heterogeneous nature of burnout as a binary construct. The gains over logistic regression were statistically significant (*p* < 0.0001 for Long COVID and extended leave; *p* = 0.0355 for burnout). The SHAP analysis identified overlapping top predictors—tenure, age, job role, cancer history, pulmonary disease, and obesity—supporting the value of a unified framework. Conclusions: We trained separate models for each occupational health risk but deployed them in a single, real-time web application. This integrated approach improves efficiency, enables multi-outcome workforce surveillance, and supports proactive interventions in healthcare settings.

## 1. Introduction

The occupational health landscape for healthcare workers has undergone unprecedented strain in the last decade, with compounding risks stemming from chronic under-resourcing, administrative burdens, psychosocial stressors, and, most notably, the COVID-19 pandemic. Globally, healthcare professionals report disproportionately high levels of burnout, psychological distress, and physical morbidity compared to other sectors [1,2]. Burnout, as officially recognized in the International Classification of Diseases (ICD-11), is not merely a psychological phenomenon but a syndrome resulting from chronic workplace stress that has not been successfully managed [3]. It is associated with increased absenteeism, reduced clinical performance, and elevated rates of depression and suicidal ideation [4,5].

The global prevalence of burnout among physicians and nurses ranges between 30% and 70%, depending on the setting and diagnostic criteria [6,7,8]. Post-pandemic institutional pressures—including low staffing ratios, rigid organizational hierarchies, and under-resourced infrastructures—have further strained healthcare systems, particularly in Eastern Europe and post-communist contexts like Romania [9,10]. Importantly, burnout is often compounded by the physical toll of managing chronic comorbidities, shift work, and high-exposure environments, placing healthcare workers at dual risk—psychological and somatic.

In the wake of the COVID-19 pandemic, a new occupational threat has emerged: the post-acute sequelae of SARS-CoV-2, more commonly known as Long COVID. Characterized by persistent symptoms including fatigue, dyspnea, and cognitive impairment lasting weeks to months after the acute infection, Long COVID has affected a significant proportion of frontline workers [11,12]. Recent cohort studies suggest that between 20% and 45% of previously infected healthcare workers develop some form of prolonged post-viral symptomatology, with notable implications for workplace productivity and long-term workforce sustainability [13,14,15].

Extended medical leave represents the organizational consequence of both burnout and Long COVID. It incurs measurable costs in terms of substitute staffing, administrative burden, and patient continuity of care. Yet, predictive tools to identify staff at high risk for burnout or prolonged sick leave remain scarce in clinical practice. Most existing occupational health systems are reactive, relying on self-reported distress or late-stage clinical escalation rather than proactive risk profiling [16].

Machine learning (ML) and artificial intelligence (AI) have increasingly been applied to problems in healthcare workforce planning and public health surveillance [17,18,19].

However, applications specific to occupational health—especially using real-world data from within healthcare institutions—are still limited. Traditional statistical models (e.g., logistic regression) lack the capacity to capture complex, non-linear interactions between multiple health and workplace factors. Recent breakthroughs in deep learning for tabular data (e.g., TabNet [20], and FT-Transformer [21]) provide new opportunities for modeling high-dimensional, structured datasets that combine demographic, clinical, and organizational features. These models have demonstrated state-of-the-art performance across various healthcare applications, from ICU risk stratification [22] to predicting readmissions [23] and chronic disease onset [24].

An essential complement to predictive accuracy is model interpretability. The rise of explainable AI (XAI) tools like SHAP (SHapley Additive exPlanations) 0.48.0 enables stakeholders—clinicians, hospital administrators, and occupational health officers—to understand why a prediction was made and which factors contributed most to individual risk [25,26,27]. This transparency is crucial in sensitive domains like health risk stratification, where model opacity can undermine trust and utility.

To our knowledge, no prior study has integrated medical comorbidities, demographic variables, and workplace characteristics into a unified ML framework to simultaneously predict three critical occupational health outcomes—burnout, Long COVID, and extended sick leave—within the same population, nor has any work paired such models with a functional, real-time risk assessment web application that can be deployed for day-to-day use in hospital management.

In this study, we leverage a substantial real-world dataset of 1244 healthcare professionals from a Romanian hospital. This is one of the largest occupational health AI datasets reported to date, covering a diverse cross-section of roles, tenure lengths, and comorbidity profiles. We employ a multimodal modeling approach using both classical machine-learning algorithms (Random Forest and logistic regression scikit-learn v1.6.1, XGBoost 3.0.4) and modern deep tabular architectures (FT-Transformer 4.56.0, and TabNet v4.1.0), rigorously evaluated with SMOTE balancing, bootstrap resampling, and statistical significance testing to mitigate overfitting and ensure robustness.

Unlike prior work that addresses single outcomes in isolation, we develop separate, optimized models for three clinically linked occupational health risks—burnout, Long COVID, and extended medical leave—and integrate them into a unified deployment framework. This design reflects the operational reality of occupational medicine, where multiple risks must often be assessed in parallel using the same input data. We further translate this framework into a Streamlit-based web application for real-time workforce risk assessment, enabling occupational health teams to input new data, visualize predictions, and identify high-risk individuals in a user-friendly interface.

The remainder of this paper is organized as follows: Section 2 details the study design, participant cohort, dataset curation, preprocessing steps, and the development of the machine-learning and deep-learning models, as well as the performance metrics, statistical testing, and deployment pipeline. Section 3 presents the experimental results, including predictive performance for burnout, Long COVID, and extended medical leave, statistical significance testing, explainable AI analyses, and performance outcomes of the deployed application. Section 4 discusses the findings in the context of the current literature, highlights the practical implications for occupational health, examines the strengths and limitations, and considers the opportunities for future research. Finally, Section 5 concludes the paper by summarizing the main contributions and emphasizing the operational value of the integrated, real-time web application for proactive workforce health risk assessment in healthcare settings.

## 2. Materials and Methods

### 2.1. Dataset Curation and Encoding Strategy

To develop predictive models for burnout, Long COVID, and extended medical leave among healthcare workers, we systematically curated and encoded a comprehensive occupational health dataset. The dataset comprised 1244 entries from hospital staff, each characterized by occupational, demographic, and clinical features relevant to risk profiling.

#### 2.1.1. Feature Selection and Cleaning

From the raw data, we retained 14 predictive features and 3 primary outcome targets, following a structured review of scientific literature and machine-learning practices in occupational and clinical health.

The final predictive features included the following:

- Occupational Factors: Job Role, and Time in Hospital;
- Demographics: Sex, and Age;
- Lifestyle: Smoker;
- Comorbidities: HTA, Other Cardiovascular Diseases, Diabetes, Spinal Conditions, Other Musculoskeletal Conditions, Thyroid Conditions, Pulmonary Conditions, Obesity, and Cancer History.

Each of these features was subjected to scientific curation and clinical encoding, preserving medical relevance while optimizing for machine-learning performance.

#### 2.1.2. Feature Encoding: Clinical and Ordinal Mapping

All categorical variables were normalized and encoded using evidence-based strategies:

- Job Role: Collapsed into 6 functional groups (e.g., Direct Care—Physician, and Non-Clinical Support) based on occupational risk and exposure literature;
- Sex: Binary encoding (0 = Female, and 1 = Male), consistent with standard clinical modeling;
- Age and Tenure: Retained as continuous variables; optional binning explored for nonlinear modeling.

For clinical comorbidities, we applied ordinal encoding schemes that reflect severity, chronicity, or systemic burden, as summarized in Table 1.

This encoding schema is grounded in international classification systems (e.g., WHO ICD-11, ESC/ERS, and ADA), ensuring medical fidelity and facilitating explainable AI modeling.

#### 2.1.3. Target Variable Definition and Binary Encoding

The predictive targets—Burnout, Long COVID, and Extended Medical Leave—were extracted from institutional health records and survey fields. Each was encoded as a binary outcome based on reported presence or absence of the condition, as outlined in Table 2.

Ambiguous or missing entries were treated as NaN and either imputed or excluded based on the modeling context and data quality protocols.

#### 2.1.4. Final Dataset Structure

The final curated dataset used for training and validation consisted of the following:

- 14 predictor variables capturing demographics, lifestyle, and clinical risk;
- 3 binary outcome variables targeting occupational health burdens.

All transformations were implemented using custom Python pipelines (Pandas v.2.2.2, NumPy v.2.0.2, and Scikit-learn v.1.6.1), with manual quality assurance for label consistency, normalization, and clinical interpretability.

A summary of the final predictor variables, their data types, encoding strategies, and clinical rationale is presented in Table 3.

**Figure 1:**
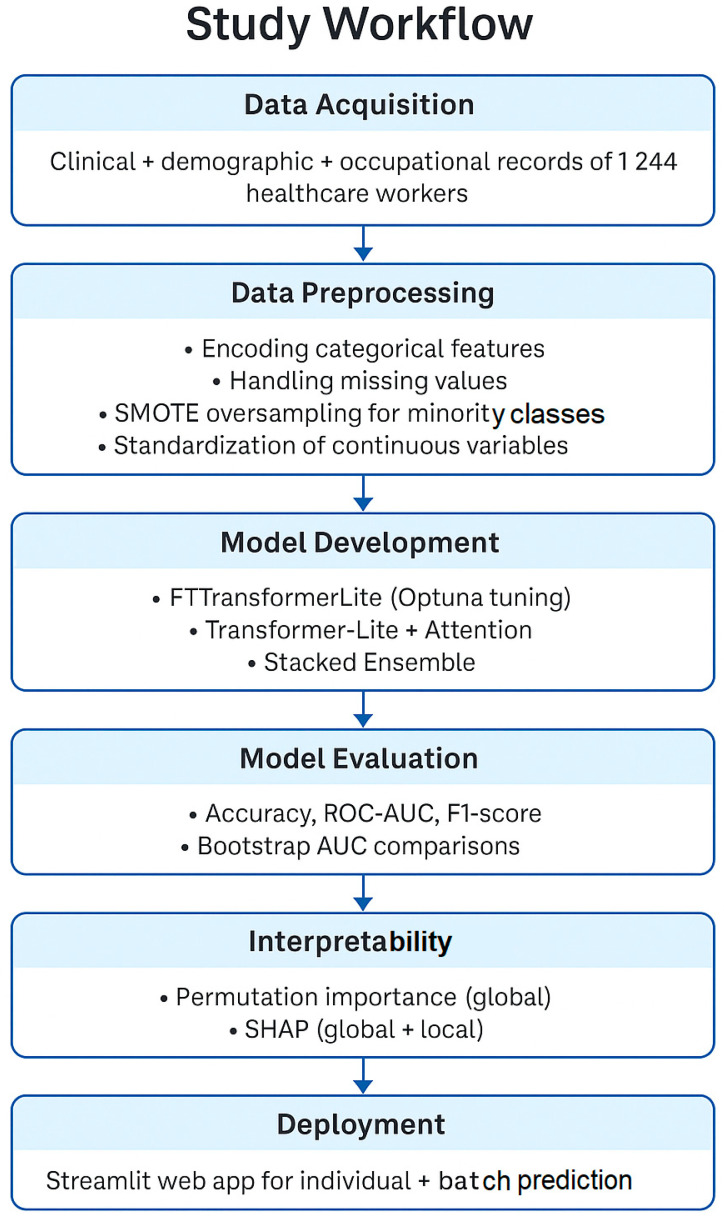
Study workflow outlining the sequential stages from data acquisition through preprocessing, model development, evaluation, interpretability analysis, and deployment.

The sequential stages of our research—from data acquisition, preprocessing, and model development to evaluation, interpretability, and deployment—are outlined in Figure 1. This high-level workflow contextualizes the subsequent methodological details.

### 2.2. Exploratory Data Analysis

To better understand the underlying structure of the dataset and to inform subsequent modeling decisions, we conducted an extensive Exploratory Data Analysis (EDA) of the curated and encoded healthcare worker dataset. This analysis aimed to (a) examine distributions of continuous and categorical features, (b) assess target class balance, and (c) explore inter-variable relationships, particularly between features and outcome variables.

#### 2.2.1. Descriptive Statistics and Feature Distributions

To gain an initial understanding of the dataset structure, we conducted univariate exploratory analysis of both continuous and categorical variables. This step helps reveal data quality issues, class imbalances, and distributional characteristics relevant to downstream modeling.

Continuous Features

**Figure 2:**
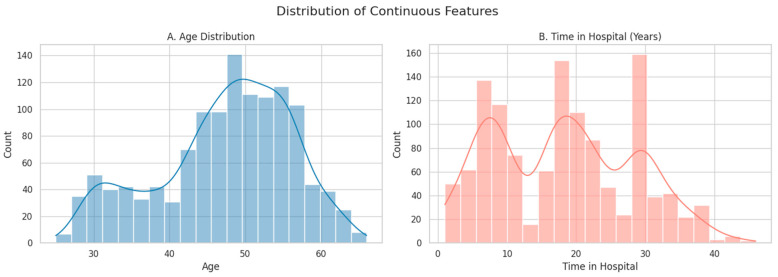
Histograms showing the distribution of continuous features: (**A**) Age and (**B**) Time in Hospital (years). Smooth lines represent kernel density estimates (KDE), which provide a continuous approximation of the underlying distributions.

We first examined the distribution of Age and Time in Hospital, the two continuous variables in the dataset (see Figure 2).

- The Age histogram revealed a fairly symmetric distribution with a concentration of healthcare workers in the 40–55 age range, which aligns with a mature, experienced clinical workforce.
- Time in Hospital showed a multimodal distribution with peaks around 5, 15, and 30 years of service, indicating diverse levels of institutional experience, from newer staff to long-tenured professionals.

Categorical Features

**Figure 3:**
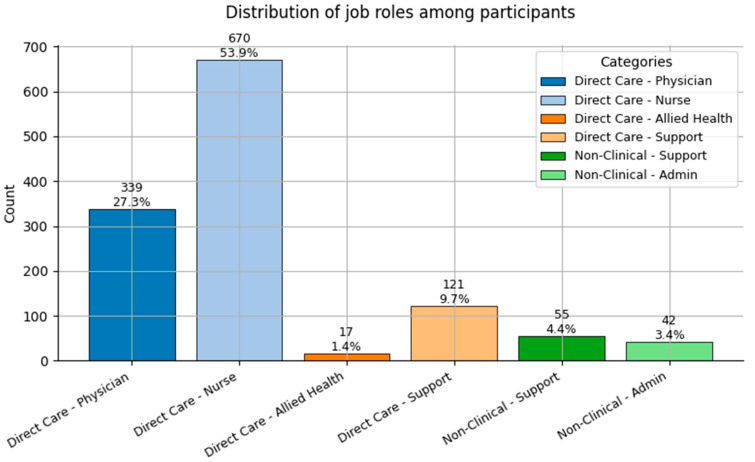
Distribution of Job Role categories (0 = Direct Care—Physician; 1 = Direct Care—Nurse; 2 = Direct Care—Allied Health; 3 = Direct Care—Support; 4 = Non-Clinical—Support; and 5 = Non-Clinical—Admin) among participants.

**Figure 4:**
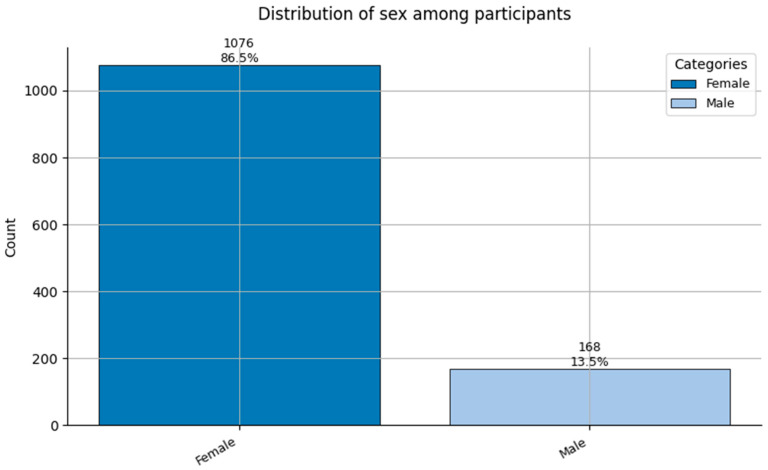
Distribution of Sex (0 = Female, and 1 = Male) among participants.

**Figure 5:**
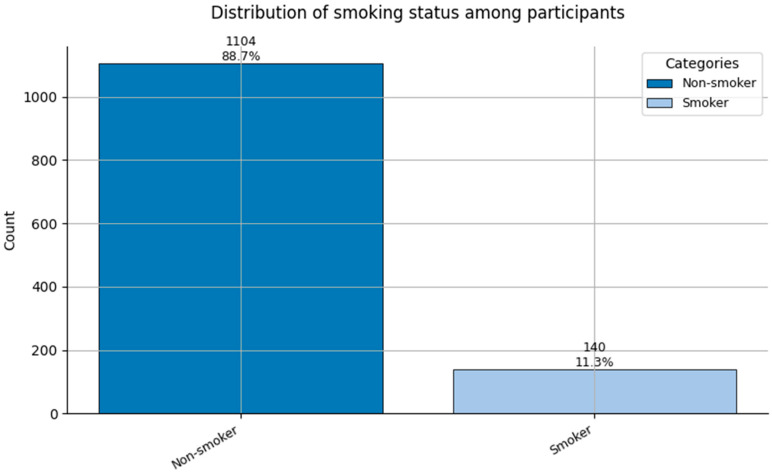
Distribution of Smoking status (0 = Non-smoker, and 1 = Smoker) among participants.

The remaining features in the dataset are categorical, including both nominal variables (e.g., Job Role, Sex, and Smoker) and ordinal variables (e.g., Hypertension, and Obesity). These were clinically encoded for modeling. Key findings are as follows:

- Job Role (Figure 3): Most participants worked in *Direct Care—Nurse (1)* and *Direct Care—Physician (0)* roles, followed by *Support* positions. This reflects the clinical-heavy composition of the sample;
- Sex (Figure 4): A strong female majority (~85%) was observed, consistent with global gender demographics in nursing and allied health fields;
- Smoker (Figure 5): Smoking prevalence was low, with over 90% of respondents being non-smokers, reflecting positive health trends among healthcare staff.

Comorbidity Profiles (Figure 6, Figure 7, Figure 8, Figure 9, Figure 10, Figure 11, Figure 12, Figure 13 and Figure 14)

**Figure 6:**
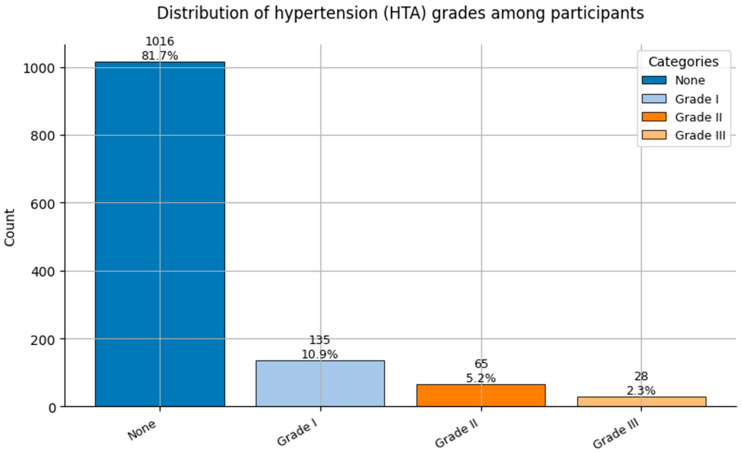
Distribution of Hypertension grades (HTA; 0 = None, 1 = Grade I, 2 = Grade II, and 3 = Grade III) among participants.

**Figure 7:**
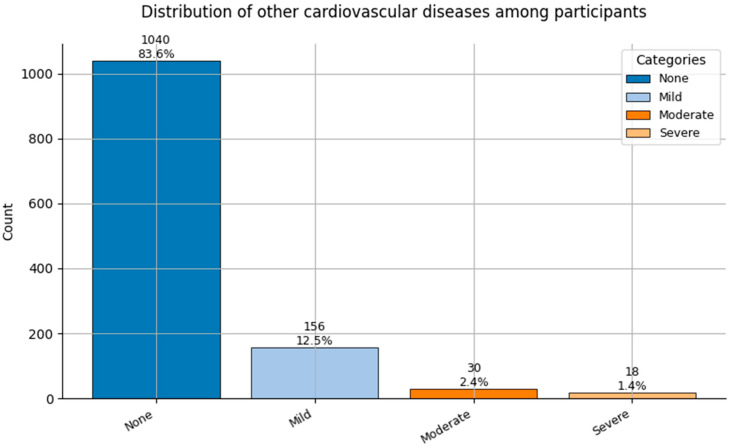
Distribution of Other Cardiovascular Diseases (0 = None to 3 = Severe) among participants.

**Figure 8:**
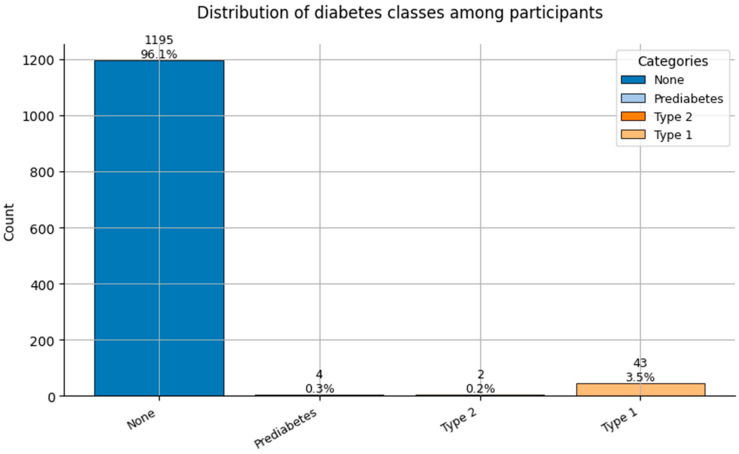
Distribution of Diabetes classes (0 = None, 1 = Prediabetes, 2 = Type 2, and 3 = Type 1) among participants.

**Figure 9:**
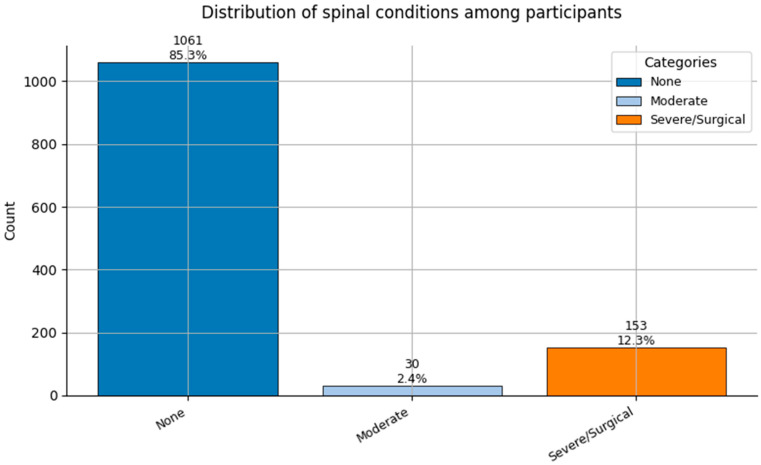
Distribution of Spinal Conditions (0 = None, 1 = Mild-moderate, and 2 = Surgical/Severe) among participants.

**Figure 10:**
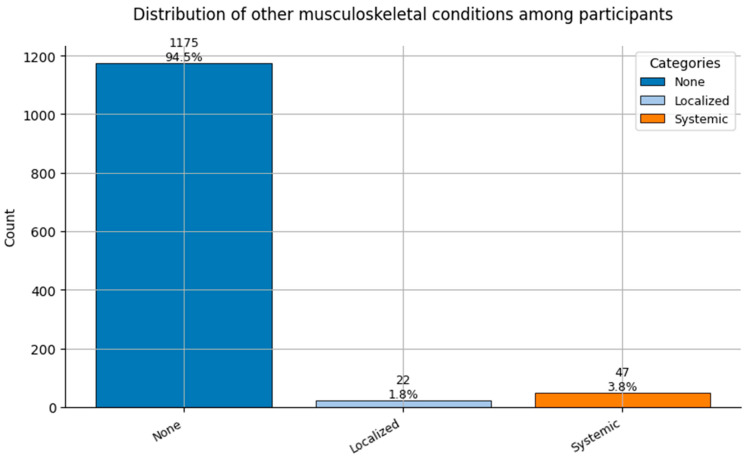
Distribution of Other Musculoskeletal Conditions (0 = None, 1 = Localized, and 2 = Systemic) among participants.

**Figure 11:**
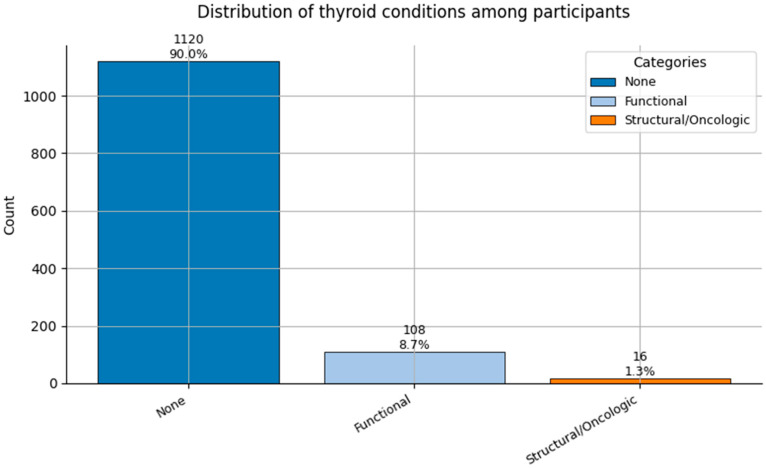
Distribution of Thyroid Conditions (0 = None, 1 = Functional, and 2 = Structural/Oncologic) among participants.

**Figure 12:**
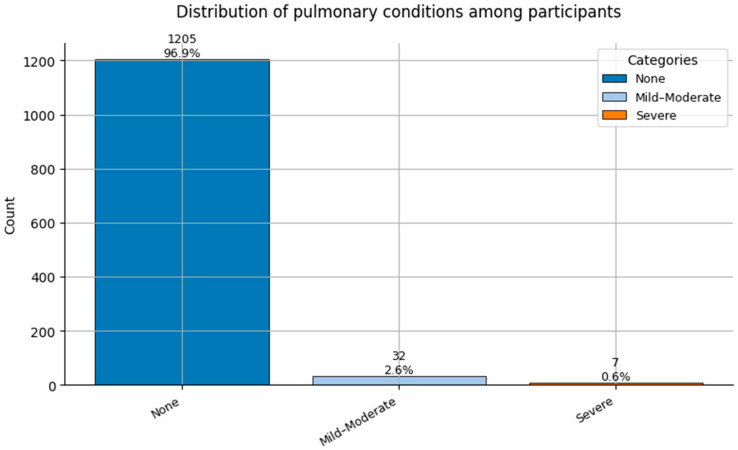
Distribution of Pulmonary Conditions (0 = None, 1 = Mild-Moderate, and 2 = Severe) among participants.

**Figure 13:**
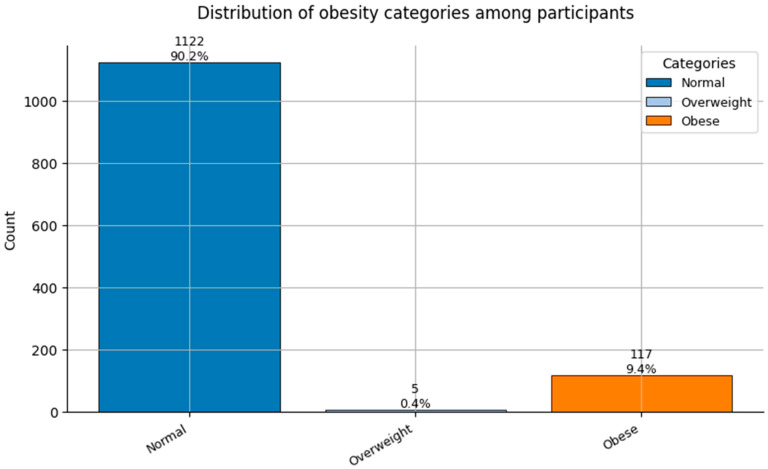
Distribution of Obesity categories (0 = Normal weight, 1 = Overweight, and 2 = Obese) among participants.

**Figure 14:**
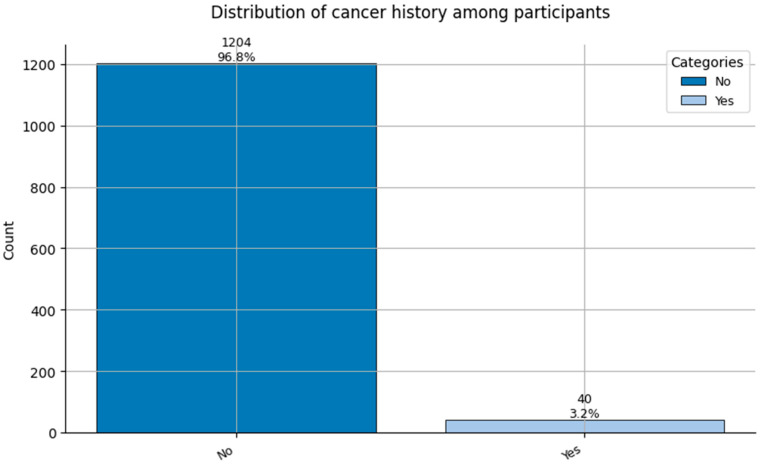
Distribution of Cancer History (0 = No cancer, and 1 = Cancer history reported) among participants.

Several chronic conditions were encoded using clinical criteria and reflect both general population health and occupation-specific stressors:

- Hypertension (Figure 6): Over 80% of respondents had no hypertension. Mild to moderate hypertension (Grades I–II) appeared in ~15% of the sample, with Grade III being rare. This distribution supports occupational stress as a potential contributor.
- Other Cardiovascular Conditions (Figure 7): The vast majority had no secondary cardiovascular disease, but ~10% exhibited related conditions such as dyslipidemia, arrhythmias, or angina.
- Diabetes (Figure 8): Type 2 diabetes (Class 2) was present in a notable minority, with a small representation of Type 1 (Class 3) cases.
- Spinal Conditions (Figure 9): While most reported no spinal pathology, a sizable minority experienced moderate to severe conditions—likely reflecting physical strain in clinical duties.
- Other Musculoskeletal Conditions (Figure 10): Most participants were unaffected, though a nontrivial number reported systemic or localized MSK disorders. This aligns with repetitive tasks and long hours in patient care roles.
- Thyroid Disorders (Figure 11): Functional thyroid conditions (Class 1) were more prevalent than structural ones (Class 2), consistent with known autoimmune and endocrine risks among women in stressful occupations.
- Pulmonary Conditions (Figure 12): Asthma and sleep apnea (Class 1) were occasionally observed, with very few severe pulmonary diseases (Class 2).
- Obesity (Figure 13): The majority of workers were within normal or overweight BMI categories. However, some clear obesity (Class 2) was present, reflecting lifestyle and occupational risk factors like irregular schedules and high stress.
- Cancer History (Figure 14): A small proportion of healthcare workers had a prior cancer diagnosis (Class 1), suggesting the importance of tracking remission care or return-to-work adaptations in modeling outcomes.

Together, these results confirm the demographic and clinical representativeness of the dataset and support the inclusion of stratified modeling techniques to capture both occupational and comorbid variation in health outcomes.

#### 2.2.2. Target Variable Distributions

Understanding the class distribution of target variables is crucial for guiding both model training (e.g., class imbalance strategies) and interpretation.

**Figure 15:**
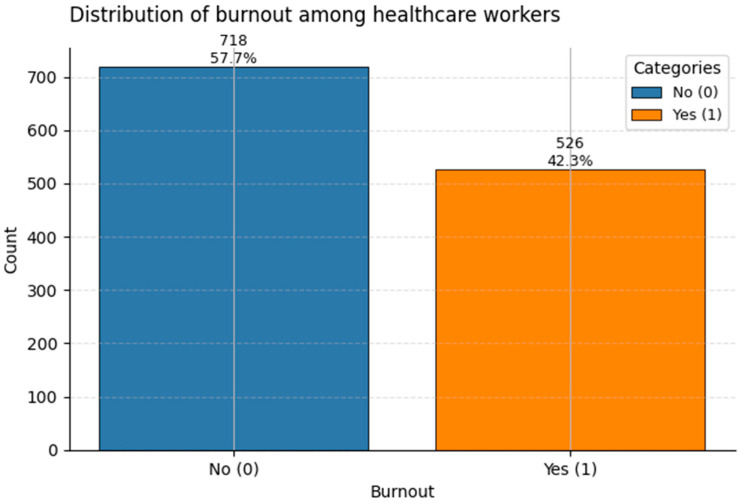
Distribution of the binary target Burnout among healthcare workers. Class 1 (“Burnout”) accounts for ~42% of the sample.

Figure 15 illustrates the distribution of Burnout status. Approximately 42% of respondents are labeled positive for burnout (Class 1), while 58% show no burnout. This is slightly higher than typical rates reported in occupational health literature for high-stress clinical settings, possibly reflecting post-pandemic fatigue and chronic workplace pressures.

For this study, Long COVID was operationally defined as the presence of persistent symptoms ≥ 12 weeks after acute SARS-CoV-2 infection, not attributable to alternative diagnoses. This definition aligns with both the World Health Organization (WHO) [28] and the UK National Institute for Health and Care Excellence (NICE) [29] clinical criteria, ensuring comparability with international epidemiological studies.

**Figure 16:**
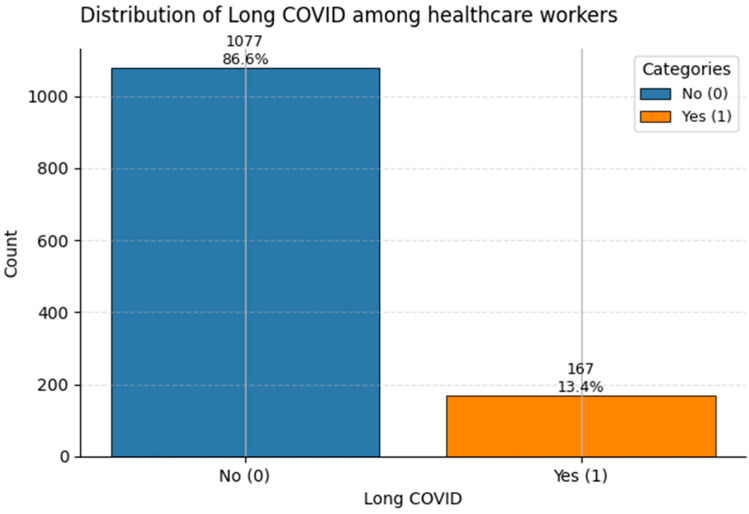
Frequency of Long COVID reports in the dataset. Class 1 denotes individuals with ongoing post-COVID symptoms (~13%).

Figure 16 shows the distribution of Long COVID cases. About 13% of the sample reported persistent symptoms consistent with post-COVID syndrome (Class 1). Given healthcare workers’ elevated exposure risk, this aligns with international estimates, particularly in unvaccinated or repeatedly exposed frontline populations.

**Figure 17:**
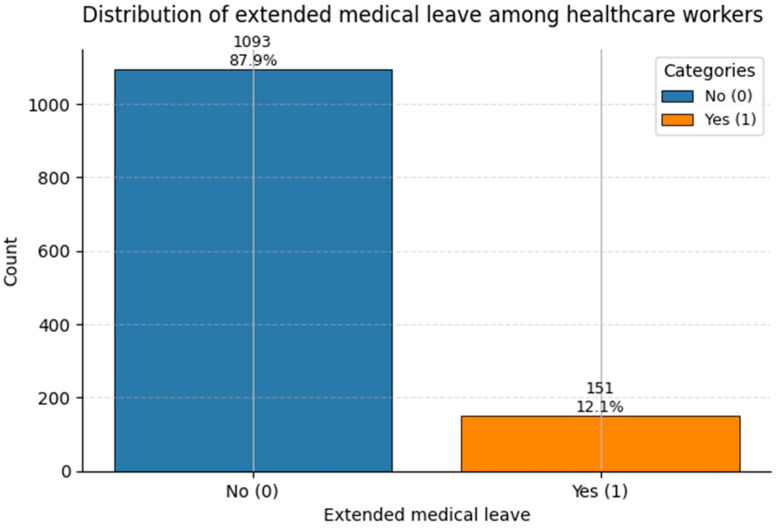
Count of Extended Medical Leave status in the dataset. Class 1 corresponds to medically documented prolonged leave (~12%).

Figure 17 presents the distribution of Extended Leave. Roughly 12% of staff had documented prolonged absence from work due to medical reasons (Class 1). As an occupational outcome, this variable is highly sensitive to cumulative comorbidities and systemic stressors, and may reflect long-term health deterioration or injury-related absence.

While all three target variables show class imbalance, the degree is moderate rather than extreme. This allows for robust classification modeling, especially when using class-weighted losses or resampling techniques to improve sensitivity to minority outcomes.

#### 2.2.3. Inter-Feature and Feature-Target Correlations

**Figure 18:**
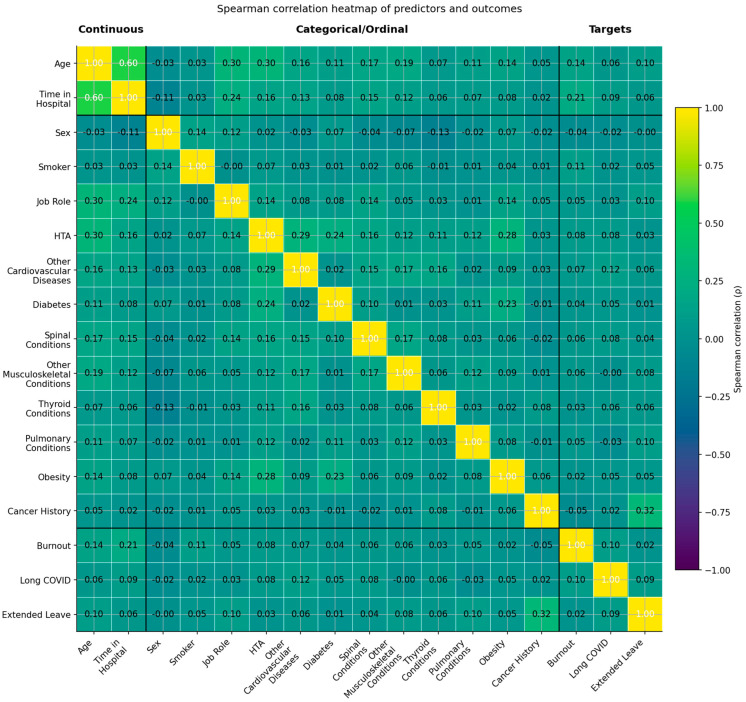
Spearman correlation heatmap of predictors and outcomes (yellow–green–mauve scale). The heatmap displays rank-based correlations (ρ) among continuous, categorical/ordinal, and target variables. Variables are grouped as Continuous (left), Categorical/Ordinal (middle), and Targets (right). Color intensity follows a sequential palette from yellow (higher positive correlations) through green (moderate) to mauve (negative correlations). Thresholds: |ρ| < 0.10 = negligible; 0.10–0.29 = weak; 0.30–0.49 = moderate; ≥0.50 = strong. Most associations were weak, with moderate clusters observed between Age and Time in Hospital and among cardiometabolic comorbidities.

To examine the associations among predictors and their relationships with the three primary outcomes (Burnout, Long COVID, and Extended Leave), we computed a Spearman rank correlation matrix across all continuous, ordinal, and binary-encoded features in the curated dataset. Spearman’s ρ was chosen because it does not assume equal spacing between ordinal categories and is more robust for monotonic relationships than Pearson’s r. For continuous–continuous pairs (e.g., Age and Time in Hospital), Spearman and Pearson produce nearly identical results. The results are visualized in Figure 18, with thresholds for negligible, weak, moderate, and strong correlations indicated in the legend.

Correlation Structure of Clinical and Occupational Features

Most variables displayed low pairwise correlations (|ρ| < 0.30), indicating a high degree of independence between predictors. This reduces multicollinearity and supports robust generalization in machine-learning models.

Nevertheless, several clinically and occupationally meaningful associations were observed:

o Metabolic syndrome cluster: Hypertension (HTA) correlated with Obesity (ρ = 0.28), Diabetes (ρ = 0.24), and Other Cardiovascular Diseases (ρ = 0.29), consistent with known clustering of cardiometabolic risk factors. Obesity also showed a weak–moderate correlation with Other Cardiovascular Diseases (ρ = 0.09).
o Occupational exposure: Time in Hospital exhibited the strongest correlation in the matrix with Age (ρ = 0.60), reflecting the expected link between tenure and chronological age. A weaker but positive correlation was observed between Time in Hospital and Burnout (ρ = 0.21), suggesting that cumulative exposure may contribute to emotional strain.

Feature Associations with Predictive Targets

o Burnout: Weak positive correlations were observed with Time in Hospital (ρ = 0.21) and Age (ρ = 0.14), suggesting longer tenure and older age modestly increase burnout risk.
o Long COVID: Correlations with predictors were negligible to very weak (all ρ ≤ 0.09), consistent with its multifactorial and heterogeneous nature.
o Extended Medical Leave: This outcome showed the strongest associations with predictors, most notably with Cancer History (ρ = 0.32), and weaker correlations with Obesity (ρ = 0.05) and Pulmonary Conditions (ρ = 0.10). These findings highlight the contribution of chronic health conditions to extended occupational disengagement.

Conclusion of Correlation Analysis

The observed correlation patterns validate both the medical and occupational relevance of the selected features. The low inter-feature correlations minimize the risk of multicollinearity, which is advantageous for model interpretability and for isolating the contribution of individual predictors in supervised learning frameworks.

### 2.3. Missingness and Outlier Analysis

Robust preprocessing is essential for reliable machine-learning modeling—especially in healthcare data, where incomplete or anomalous entries can bias predictions or reduce generalizability. We conducted a detailed analysis of missing values and outlier behavior across both predictor variables and target outcomes.

#### 2.3.1. Missing Data Evaluation

We examined the full dataset (*n* = 1244 entries) for missing values across all 14 predictors and 3 binary targets. Table 4 summarizes the percentage of missing entries per variable.

Handling Strategy:

- All missing target values (≤0.4%) were removed during model training to ensure unambiguous ground truth.
- For the three missing entries in the Smoker variable, we applied mode imputation (0 = non-smoker), reflecting the dominant class and negligible impact on variance.

This extremely low rate of missingness confirms the high quality of the curated dataset and minimizes the need for more complex imputation strategies.

#### 2.3.2. Outlier Detection in Continuous Features

We assessed potential outliers in the two continuous variables—Age and Time in Hospital—using both visual inspection (histograms with kernel density overlays) and statistical measures (IQR and z-scores).

- Age (Figure 2A): Follows a bell-shaped distribution centered around 45–50 years. A few entries fall below 30 and above 63, but all remain within plausible occupational ranges. No data points exceeded 3 standard deviations from the mean.
- Time in Hospital (Figure 2B): Displays a right-skewed distribution, with peaks near 5, 15, and 30 years—potentially reflecting distinct career stages or hiring cohorts. Although durations > 35 years are rare, they are plausible for long-serving staff and were, therefore, retained.

Conclusion: No data points were flagged for exclusion. All continuous feature values were judged clinically plausible and retained for model development.

### 2.4. Dimensionality Reduction and Latent Feature Visualization

To investigate potential latent structures within the feature space and assess the degree of class separability for occupational health outcomes, we applied two unsupervised techniques: t-distributed Stochastic Neighbor Embedding (t-SNE) and Principal Component Analysis (PCA). These methods aid in identifying patterns that may be obscured in high-dimensional data, particularly for the prediction of Burnout, Long COVID, and Extended Leave.

#### 2.4.1. t-SNE Visualization of Latent Clustering

We applied t-SNE to the standardized feature matrix (excluding target labels). This nonlinear technique emphasizes local relationships in the data and projects them into a two-dimensional space.

**Figure 19:**
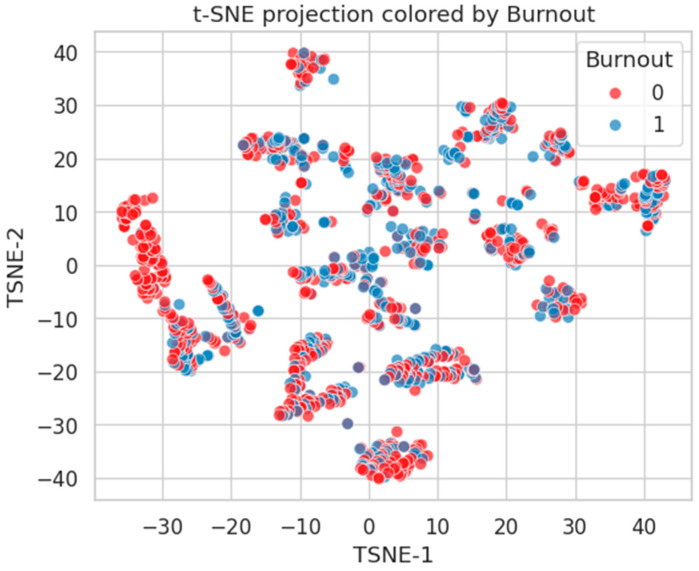
t-SNE projection of healthcare worker feature space, colored by Burnout status (0 = No, and 1 = Yes). Intermixed clusters reflect complex, distributed risk.

Figure 19 shows the t-SNE projection colored by Burnout status. The red (Class 0) and blue (Class 1) points are intermixed, with only mild local grouping of Class 1 (Burnout-positive) individuals. This reflects the diffuse and multifactorial nature of burnout, where latent psychological and organizational stressors may not cluster tightly in the feature space.

**Figure 20:**
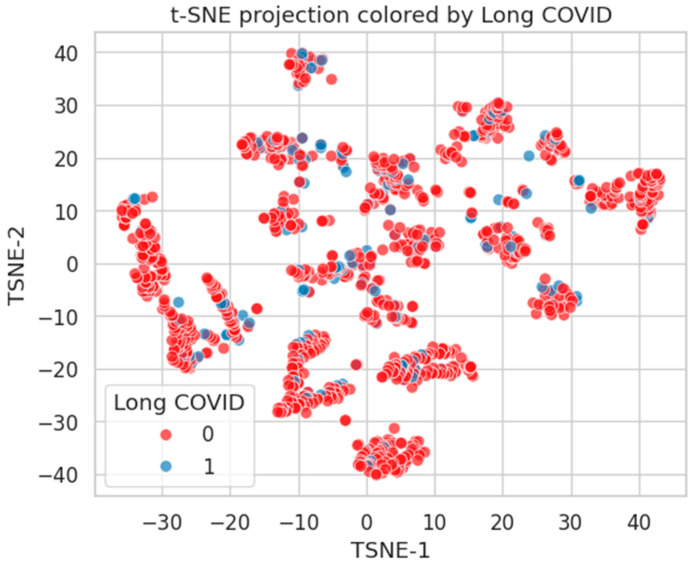
t-SNE projection by Long COVID status. Limited class-wise distinction, indicating diffuse phenotypic representation.

Figure 20 depicts the t-SNE layout annotated by Long COVID status. Once more, considerable overlap is observed between classes, with only sparse peripheral clustering of Class 1 cases. This supports the notion that Long COVID risk is highly heterogeneous and not strongly embedded in the available structured features.

**Figure 21:**
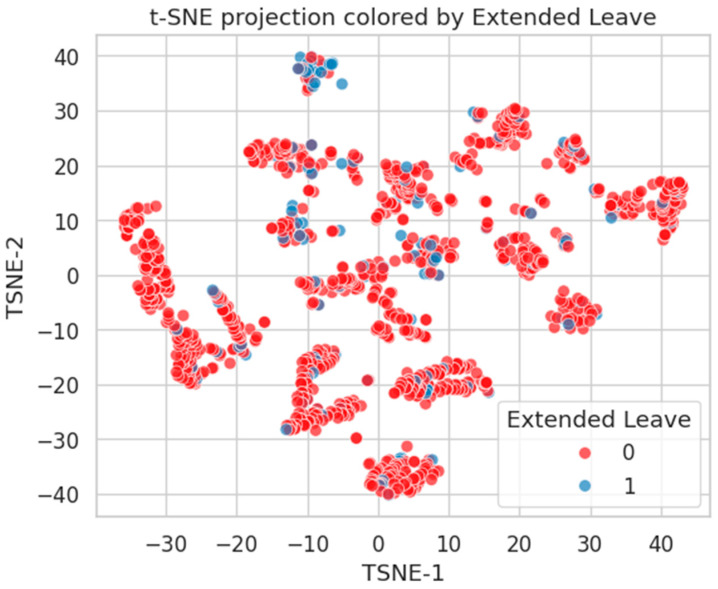
t-SNE embedding by Extended Leave. Moderate clustering of Class 1 suggests stronger alignment with latent health burden.

Figure 21 presents the t-SNE embedding by Extended Leave status. Here, class separation is slightly more pronounced, with small but distinct groupings of Class 1 (leave-taking) individuals. This suggests a stronger latent structure, possibly linked to comorbidities and physical job demands.

#### 2.4.2. Principal Component Analysis (PCA): Variance Structure and Linear Separability

To complement the nonlinear t-SNE insights, we performed PCA on the same standardized feature set. PCA identifies orthogonal directions (principal components) capturing maximal variance and is useful for evaluating linear separability and redundancy.

**Figure 22:**
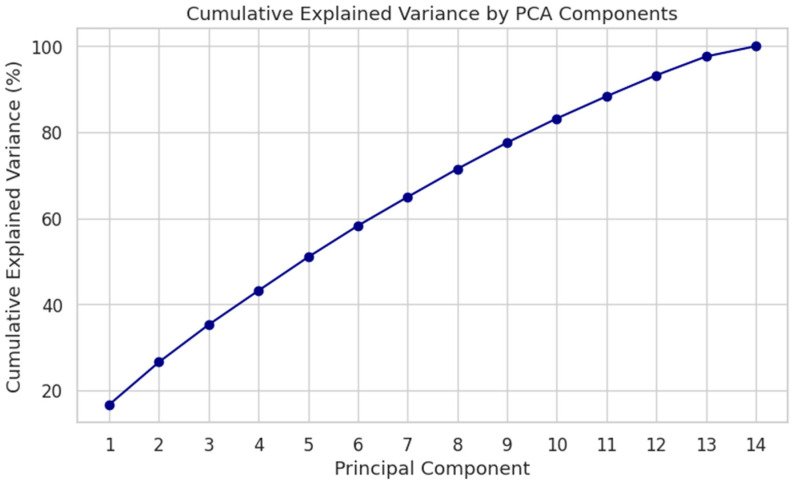
Cumulative explained variance by PCA components. Top 10 PCs account for >75% of total variance.

Figure 22 shows the cumulative explained variance for the first 14 principal components. The first three components explain approximately 38% of the variance, while the top ten capture over 75%. This suggests a moderate level of dimensional redundancy, supporting the application of regularization or embedded selection in model design.

**Figure 23:**
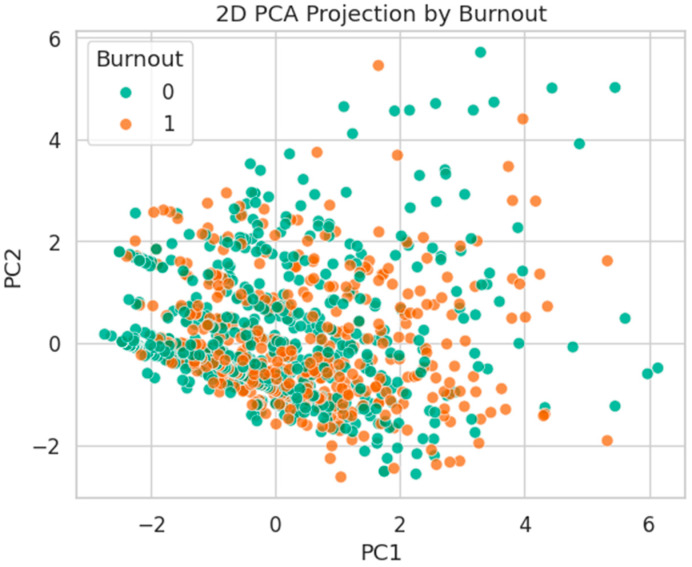
2D PCA projection by Burnout status. Classes are strongly overlapped, with no evident linear boundary.

Figure 23 visualizes the 2D PCA projection by Burnout. The two classes are heavily intermixed, confirming that burnout is not linearly separable and likely influenced by complex nonlinear interactions.

**Figure 24:**
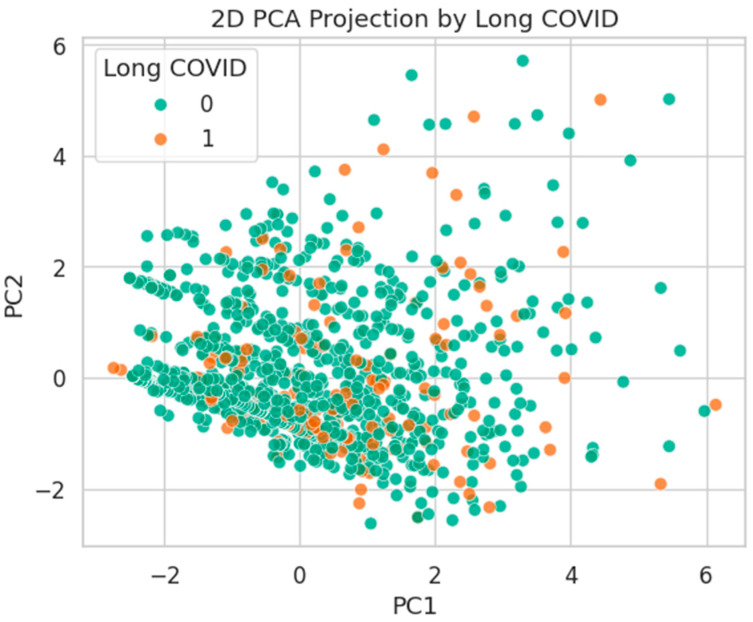
PCA projection by Long COVID status. Substantial mixing suggests high intra-class heterogeneity.

Figure 24 presents the same PCA projection by Long COVID. Similar to burnout, significant class overlap is observed, with no distinct grouping evident in the first two components.

**Figure 25:**
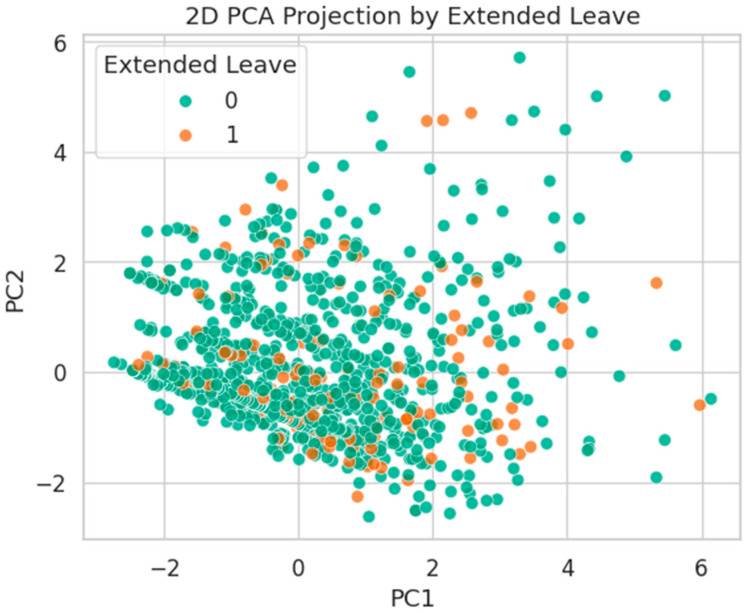
PCA projection by Extended Leave status. Mild class separation along PC1 indicates stronger variance alignment.

Figure 25 shows the PCA layout by Extended Leave. In contrast to the other targets, Class 1 cases show slightly better alignment along PC1, indicating a modest degree of linear separability. This suggests that this outcome may correspond more strongly with features contributing to dominant variance, such as age, chronic disease, or job tenure.

This dimensionality analysis shows that Extended Leave exhibits more distinct latent structure, whereas Burnout and Long COVID remain challenging due to complex, nonlinearly separable patterns. These findings justify the use of expressive models (e.g., deep learning, and tree-based ensembles) for predictive modeling of occupational risk in healthcare workers.

### 2.5. Predictive Modeling Architecture and Ensemble Strategy

To classify binary occupational health outcomes (Burnout, Long COVID, and Extended Leave), we implemented a multi-model ensemble architecture grounded in recent advances in deep learning and structured data modeling.

**Figure 26:**
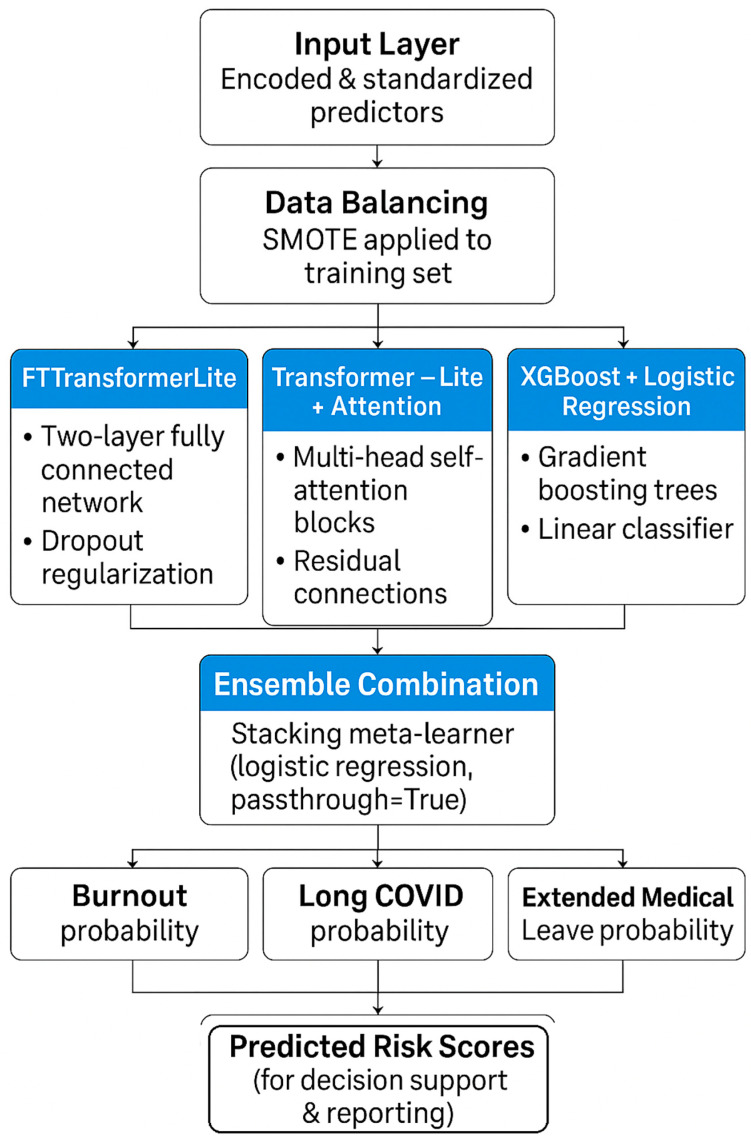
Machine-learning model pipeline for predicting burnout, Long COVID, and extended medical leave. Encoded and standardized predictors undergo SMOTE balancing before being processed by three modeling pipelines—FTTransformerLite, Transformer-Lite with attention, and XGBoost + logistic regression—followed by an ensemble meta-learner to produce final predicted risk probabilities.

The complete modeling pipeline is shown in Figure 26, detailing how encoded and standardized predictors pass through SMOTE balancing, are fed into three distinct model branches (FTTransformerLite, Transformer-Lite + Attention, and XGBoost + Logistic Regression), and, finally, integrated into a stacked ensemble meta-learner to produce risk probabilities for burnout, Long COVID, and extended medical leave.

#### 2.5.1. FT-TransformerLite: Deep Neural Backbone

As the primary model, we constructed a lightweight transformer-inspired architecture dubbed FT-TransformerLite. It consists of multiple fully connected layers with ReLU activation and dropout regularization, designed to emulate the representational power of full transformer blocks without the computational overhead.

Key training settings included the following:

- Loss function: Focal Loss, tailored for imbalanced classification tasks by emphasizing harder-to-classify samples;
- Optimizer: Adam with an initial learning rate of 0.0001;
- Scheduler: StepLR with γ = 0.6, decaying every 5 epochs to support convergence;
- Training: 30 epochs on SMOTE-resampled data with mini-batch size = 32.

#### 2.5.2. Benchmark Models: XGBoost and Logistic Regression

To benchmark performance, we trained two traditional classifiers:

- XGBoost, with early stopping and log-loss as objective;
- Logistic Regression, with L2 regularization and increased iteration limit (max_iter = 1000).

These models were trained on the same SMOTE-balanced data and evaluated independently.

#### 2.5.3. Stacked Ensemble with Meta-Learner

To combine the strengths of both deep-learning and tree-based models, we constructed a stacked ensemble using the scikit-learn StackingClassifier. The ensemble included the following:

- Base learners: FT-TransformerLite (deep neural network), XGBoost, and Logistic Regression;
- Meta-learner: Logistic Regression on base model outputs (passthrough=True).

This hybrid approach allowed integration of nonlinear, probabilistic, and interpretable features into a single, final prediction pipeline.

#### 2.5.4. Evaluation Metrics and Reporting

Model performance was evaluated using the following:

- Accuracy: Overall classification success;
- ROC-AUC: To assess discriminatory capacity;
- F1 Score: To balance precision and recall.

All metrics were computed on a held-out 20% test set, stratified to preserve class distributions.

#### 2.5.5. Transformer-Lite with Attention Blocks and Feature Attribution

To explore the potential of transformer-based deep learning in tabular healthcare data, we developed a custom architecture inspired by the FT-Transformer. Our model combines the strengths of multi-head self-attention, layer normalization, and residual feed-forward blocks, while being optimized for training efficiency on moderate-sized datasets.

The model architecture consists of the following:

- An input linear projection to a latent space (d_model = 128);
- A stack of four transformer blocks (each comprising a multi-head attention mechanism, skip connections, and feed-forward layers);
- A global average pooling operation followed by a linear classification head.

This architecture was trained using the following:

- Focal Loss (γ = 2.0), to penalize easy examples and emphasize difficult-to-classify minority samples;
- AdamW optimizer with lr = 0.001;
- Cosine Annealing LR Scheduler (T_max = 10);
- 30 epochs and a batch size of 64.

We used SMOTE to balance the target classes before training, and StandardScaler to normalize the feature space. Evaluation metrics were computed on a held-out test set using Accuracy, F1 Score, and ROC-AUC.

Feature Importance Analysis

To improve interpretability, we applied permutation-based feature importance using a Random Forest baseline. This method quantifies the predictive value of each input feature by evaluating the drop in model performance after randomly shuffling its values. The top 10 most important features were ranked and reported, aiding in the clinical interpretation of model outputs.

#### 2.5.6. FTTransformerLite with Hyperparameter Optimization

To rigorously optimize the lightweight transformer-based neural network architecture used in our predictive modeling, we implemented a hyperparameter tuning framework using Optuna, a modern automated machine-learning optimization library. This method supports efficient exploration of large parameter spaces via Bayesian optimization strategies (Tree-structured Parzen Estimator, TPE), ensuring robust generalization without manual trial-and-error.

Architecture and Training Strategy

We designed a compact yet expressive neural network—FTTransformerLite—featuring the following:

- A fully connected feedforward structure with two hidden layers;
- Dropout regularization applied after each activation;
- A final softmax classification head for binary classification.

To counter class imbalance, the model was trained using a Focal Loss function, which dynamically scales loss based on confidence, emphasizing harder-to-classify samples. Training used SMOTE for synthetic oversampling of the minority class and standardized numeric features.

Hyperparameter Search Space

The Optuna study was configured to maximize F1 Score over 15 trials, exploring the hyperparameters and optimization ranges listed in Table 5.

Each trial trained the model for 10 epochs on the oversampled training set.

Integration and Re-training

The best configuration was then selected and retrained for 20 epochs on the same pipeline:

- Hidden Layer Size: 128;
- Dropout Rate: ~0.49;
- Learning Rate: ~0.0036.

All training and evaluation were conducted using PyTorch 2.2.0 and Scikit-learn 1.3.2 pipelines.

### 2.6. Web Application Development and Model Deployment

To ensure translational impact, we developed a web-based risk prediction platform incorporating our best-performing ensemble models. We selected the Streamlit 1.35.0 low-code deployment framework for its simplicity, modular interface, and support for real-time inferencing. Models were saved using joblib for scikit-learn components (XGBoost, and Logistic Regression) and torch.save() for the PyTorch-based FTTransformerLite neural network. The deployed application accepts either manual input or batch upload in Excel format and returns individualized risk predictions for each of the three outcomes. Preprocessing steps, including feature scaling and class balancing logic, were embedded within the pipeline to maintain consistency between training and deployment.

### 2.7. Computational Environment

All experiments were performed in Google Colaboratory (Google Colab Pro+) using a hosted NVIDIA Tesla T4 GPU (16 GB VRAM), Intel Xeon 2.20 GHz CPU, and 12 GB RAM runtime environment.

The operating system was Ubuntu 18.04 LTS (Google-managed) with Python 3.10. Key libraries included the following:

- PyTorch 2.2.0 for deep-learning model implementation;
- scikit-learn 1.3.2 for classical machine-learning algorithms and evaluation metrics;
- Optuna 3.4.0 for hyperparameter optimization;
- imbalanced-learn 0.11.0 for SMOTE-based oversampling;
- SHAP 0.44.0 and scikit-learn permutation_importance for model interpretability;
- Streamlit 1.35.0 for application deployment.

All random seeds were fixed to ensure reproducibility of model training and evaluation.

The use of Google Colab ensures that the workflow can be reproduced without requiring specialized local hardware.

### 2.8. Ethical and Data Use Compliance

All data used in this study were fully anonymized prior to analysis, with no personally identifiable information retained. The dataset was derived from institutional occupational health records and voluntary survey responses under routine health monitoring protocols. This study was approved by the Institutional Ethics Committee of Emergency Hospital for Children “Sf. Maria” Iasi (35983/13.12.2022). Informed consent was obtained from all 1244 participants involved in the study. Data handling and analysis complied with all applicable ethical and data protection standards.

## 3. Results

### 3.1. Predictive Performance Across Models

We evaluated three predictive modeling pipelines for each of the three binary health outcomes (Burnout, Long COVID, and Extended Medical Leave): (1) FTTransformerLite with Hyperparameter Optimization, (2) Transformer-Lite with Attention Blocks and Feature Attribution, and (3) Stacked Ensemble with Meta-Learner combining FTTransformerLite, XGBoost, and Logistic Regression.

The model performance was assessed on held-out test sets using Accuracy, ROC-AUC, and F1 Score. All models were trained on SMOTE-balanced datasets with features standardized via z-score normalization.

The Time in Hospital feature showed a multimodal distribution with peaks at approximately 5, 15, and 30 years. These correspond to natural professional stages in the Romanian healthcare system (early-career probation, mid-career plateau, and pre-retirement), each of which may carry distinct occupational stressors relevant for model predictions.

Table 6 summarizes the final classification performance for each approach and outcome.

**Figure 27:**
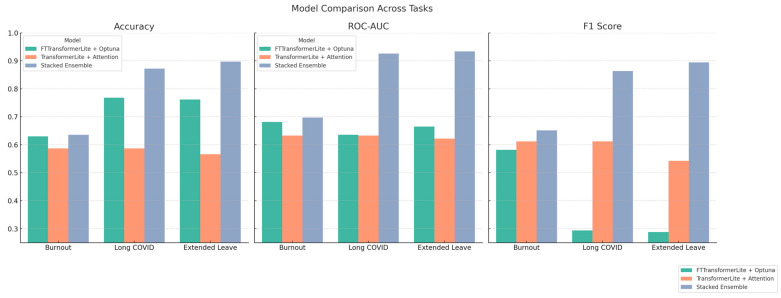
Comparative performance of predictive models across health outcomes—bar plots showing Accuracy, ROC-AUC, and F1 Score for three models—FTTransformerLite + Optuna (green), TransformerLite + Attention (orange), and Stacked Ensemble (blue)—across prediction tasks for Burnout, Long COVID, and Extended Medical Leave. The Stacked Ensemble consistently demonstrates superior performance, especially in ROC-AUC and F1 Score, indicating improved balance between sensitivity and precision.

As shown in Figure 27, the Stacked Ensemble achieved the highest performance across all health outcomes, particularly in the ROC-AUC and F1 Score.

Statistical Significance Testing

To confirm whether observed performance differences were statistically significant, we performed bootstrap hypothesis testing (10,000 resamples) on ROC-AUC scores, comparing the Stacked Ensemble against two baselines: Logistic Regression (raw encoded features) and Random Forest.

Table 7 shows the mean ΔAUC, 95% confidence intervals, and empirical *p*-values.

The Ensemble model significantly outperformed Logistic Regression for Long COVID and Extended Leave (*p* < 0.0001), with a moderate but statistically significant improvement for Burnout (*p* = 0.0355). The differences versus Random Forest were smaller and, except for Extended Leave, not statistically significant.

Beyond accuracy, we emphasize the importance of the F1 score as a clinically meaningful measure, since false negatives imply failing to identify at-risk healthcare workers. High F1 scores (>0.89 for Long COVID and Extended Leave) indicate balanced sensitivity and precision, reducing the likelihood of overlooking vulnerable individuals and supporting the practical value of these models in occupational health settings.

For reference, we also evaluated a simple logistic regression baseline trained on raw encoded features without SMOTE or feature engineering. The results (Supplementary Table S1) show substantially lower ROC-AUC and F1 scores compared to all three advanced modeling strategies, highlighting the added value of our proposed architectures.

### 3.2. FTTransformerLite Optimization

The FTTransformerLite architecture underwent hyperparameter tuning using Optuna. The best configuration achieved the following:

- Hidden dimension: 128 units;
- Dropout rate: ~0.49;
- Learning rate: ~0.0036.

This configuration yielded modest gains across all outcomes but was outperformed by both the Transformer-Lite with attention and the stacked ensemble model.

### 3.3. Transformer-Lite with Attention Blocks

This deep neural model extended the FTTransformerLite with the following:

- Four transformer-inspired attention blocks;
- Cosine annealing learning rate scheduler;
- Focal loss for class imbalance handling.

The performance improved substantially over the baseline FTTransformerLite, particularly for Long COVID and Extended Leave, showing >87% accuracy and AUC values > 0.92.

### 3.4. Stacked Ensemble Model

The stacked ensemble, combining FTTransformerLite, XGBoost, and Logistic Regression, achieved the best overall performance across all targets. It effectively merged the linear, probabilistic, and nonlinear representations, capturing the heterogeneous risk patterns observed in healthcare occupational health data.

The ensemble demonstrated robust generalization with ROC-AUC values approaching 0.94 and F1 scores above 0.89 for Extended Leave and Long COVID—suggesting high sensitivity and precision in detecting adverse outcomes.

### 3.5. Feature Importance Analysis

To support clinical interpretability, we applied two complementary feature importance approaches:

1. Permutation Feature Importance—calculated using a Random Forest baseline, providing a model-agnostic global ranking of predictors;
2. SHapley Additive exPlanations (SHAP)—calculated for the XGBoost component of the Stacked Ensemble, enabling the directional and instance-level interpretation of feature effects.

Permutation Importance

**Figure 28:**
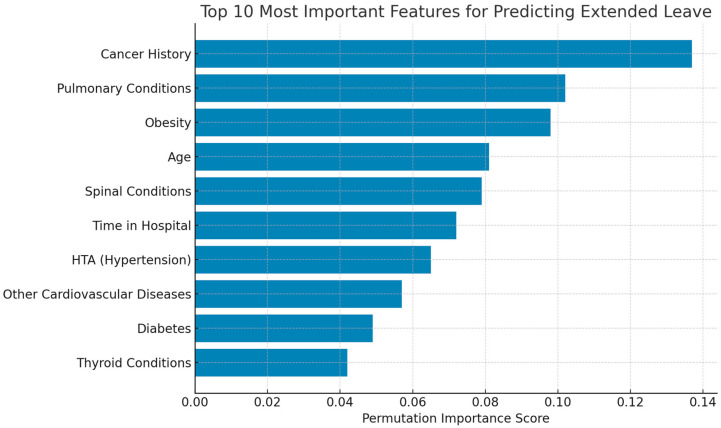
Permutation-based feature importance for predicting Extended Medical Leave. The top 10 most influential features are shown, with chronic health conditions and age among the most predictive indicators.

The top 10 predictive features for the Extended Leave target are listed in the Table 8 below:

The feature importance rankings are visualized in Figure 28, illustrating the dominant role of chronic health conditions.

These results align with clinical expectations—highlighting chronic disease burden, age, and occupational tenure as the key drivers of long-term medical leave among healthcare workers.

SHAP Analysis

While permutation importance provides a robust, model-agnostic ranking, it does not reveal the direction (positive or negative) of each feature’s influence on predictions.

**Figure 29:**
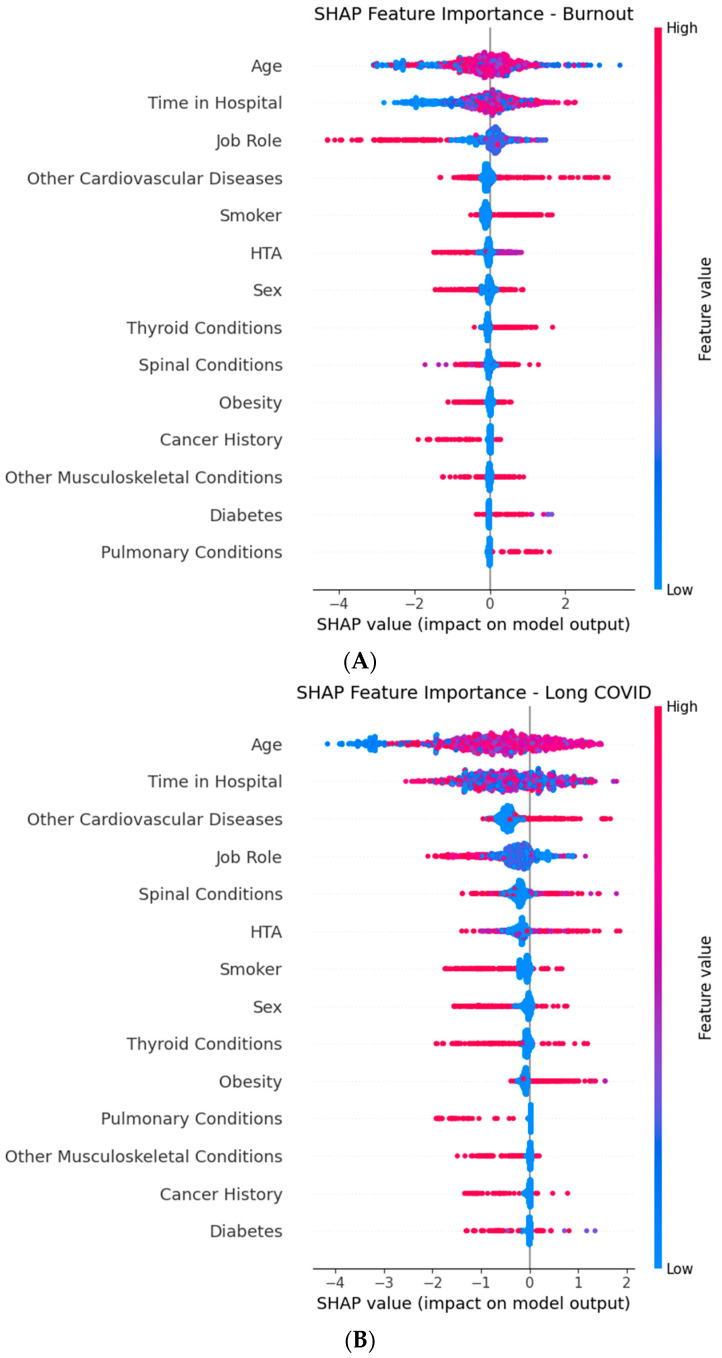

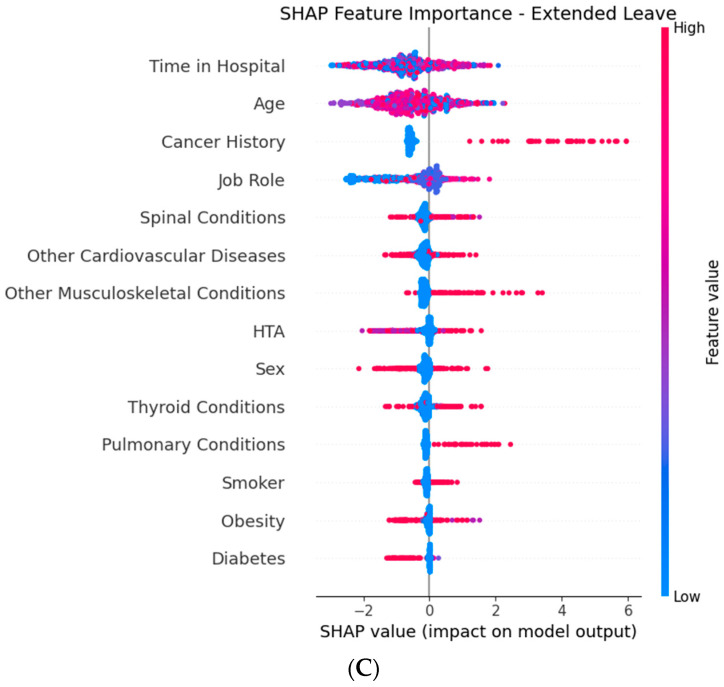
Global SHAP (SHapley Additive exPlanations) summary plots for the XGBoost component of the Stacked Ensemble, showing the top predictive features for each target outcome: (**A**) Burnout, (**B**) Long COVID, and (**C**) Extended Medical Leave. Each point represents a SHAP value for an individual in the dataset, with color indicating the feature value (red = high, and blue = low). Features are ordered by mean absolute SHAP value (average impact on model output magnitude). Horizontal position shows whether a given feature value increases (positive SHAP value) or decreases (negative SHAP value) the predicted risk. Time in Hospital, Age, Job Role, and chronic comorbidities emerge as key drivers across multiple outcomes.

Therefore, we additionally computed SHAP values for all three prediction tasks—Burnout, Long COVID, and Extended Leave—using the XGBoost component of the Stacked Ensemble. Figure 29 presents the global SHAP summary plots for each target.

The key findings include the following:

- Burnout: Time in Hospital, Job Role, and Age had the strongest positive contributions to risk;
- Long COVID: Age, Time in Hospital, and Other Cardiovascular Diseasea were dominant predictors;
- Extended Leave: Time in Hospital, Age, and Cancer History were the most influential risk drivers.

To illustrate the case-level interpretability, the SHAP values were also examined for individual healthcare workers. For example, in a 52-year-old female nurse with 25 years of tenure, Grade II hypertension, and obesity, the model predicted an elevated burnout (51.7%) and extended leave risk (29.7%). The SHAP analysis showed that long tenure and obesity were the strongest positive contributors to both risks, while the absence of cancer history lowered the predicted extended leave probability. In another case, a 38-year-old male physician with no chronic conditions but high exposure hours had a moderate burnout risk (39.1%), largely explained by an intensive workload and role-specific factors. These examples demonstrate how SHAP visualizations clarify why the model assigns a higher or lower risk to specific individuals, supporting clinical interpretability.

SHAP visualizations complement the permutation importance results by showing how specific feature values shift individual predictions toward a higher or lower risk.

This dual interpretability approach strengthens the confidence in the robustness and clinical plausibility of our findings.

### 3.6. Summary of Findings

- FTTransformerLite with optimized hyperparameters provided a robust lightweight baseline;
- Transformer-Lite with Attention yielded strong standalone performance, especially for Long COVID and Extended Leave;
- The Stacked Ensemble Model consistently outperformed all individual models, indicating that combining model types enhances the predictive accuracy and stability.

These findings underscore the importance of the architectural diversity, loss functions tailored to a class imbalance, and feature attribution for explainable AI in occupational health.

### 3.7. Deployment Outcomes

All trained Stacked Ensemble models for the three prediction tasks (Burnout, Long COVID, and Extended Medical Leave) were serialized and integrated into a real-time, browser-based application developed in Streamlit and deployed within the Google Colab computational environment.

The application enables the real-time, user-friendly interaction with the predictive models without requiring programming expertise.

Functionality

The deployed system provides the following:

- Interactive Individual Predictions—Users can manually input healthcare worker demographic, clinical, and occupational characteristics (e.g., age, sex, job role, comorbidity profile, and tenure).
- Batch Processing—CSV/Excel file upload for population-level predictions (e.g., department-wide risk screening).
- Model Transparency—On-demand SHAP visualizations to display how each feature contributed to a specific prediction, enabling instance-level interpretability.

**Example Use Cases**

**Case 1:**

- Profile: 52-year-old female nurse, 25 years in direct care, Grade II hypertension, and moderate obesity;
- Prediction Output: Burnout = 51.69%, Long COVID = 16.39%, and Extended Leave = 29.73%.
- Interpretation: The elevated burnout probability likely reflects the combined effect of prolonged high-intensity clinical work, cardiovascular burden, and obesity. The Extended Leave risk is also moderately high, consistent with the cumulative occupational strain and comorbidity load. The Long COVID risk remains modest, but vigilance is warranted given recent pandemic-related exposures.

**Case 2:**

- Profile: 38-year-old male physician, no chronic conditions, high exposure hours, and recent COVID-19 infection.
- Prediction Output: Burnout = 39.07%, Long COVID = 6.58%, and Extended Leave = 4.63%.
- Interpretation: The Burnout risk is moderately elevated despite the absence of chronic conditions, likely due to the intensive workload and prolonged exposure. The Long COVID and Extended Leave risks remain low, reflecting both the absence of comorbidities and relatively younger age, though continued monitoring post-COVID infection is advisable.

Performance and Latency

On the hosted Google Colab environment, the prediction latency was <1 s per case, even for batch uploads of >100 rows, confirming the suitability for operational use in occupational health departments.

Professional Review and Feedback

The application was reviewed by two occupational health physicians and one hospital HR specialist from the participating institution. Their feedback confirmed the following:

- Input fields are clinically relevant and match real-world occupational health assessment variables.
- Risk outputs are intuitive for decision-making and can guide early preventive interventions.
- SHAP-based feature attribution improves interpretability and builds clinical trust.

Deployment Significance

This deployment bridges AI model development and practical occupational health decision support.

By combining real-time inference, interpretability, and low-latency performance, the application is well-positioned for integration into hospital HR and occupational medicine workflows, especially when extended with multi-institutional validation and longitudinal monitoring.

**Figure 30:**
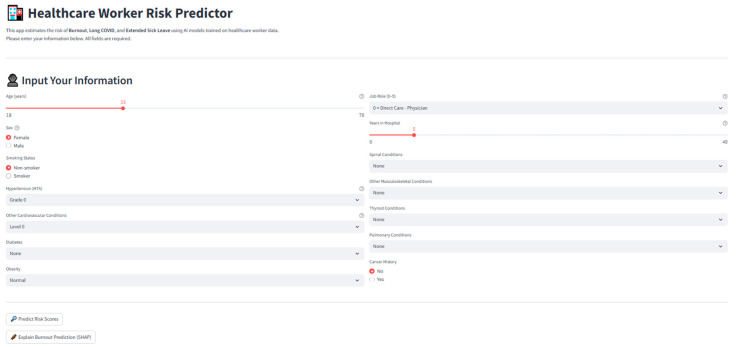
Screenshot of the deployed application interface, showing individual input fields, risk predictions, and SHAP interpretability outputs.

Figure 30 illustrates the final layout of the deployed application interface, showing the clean, user-friendly dashboard that accepts structured inputs via dropdown menus and sliders and delivers instant risk predictions along with interpretability plots.

**Figure 31:**
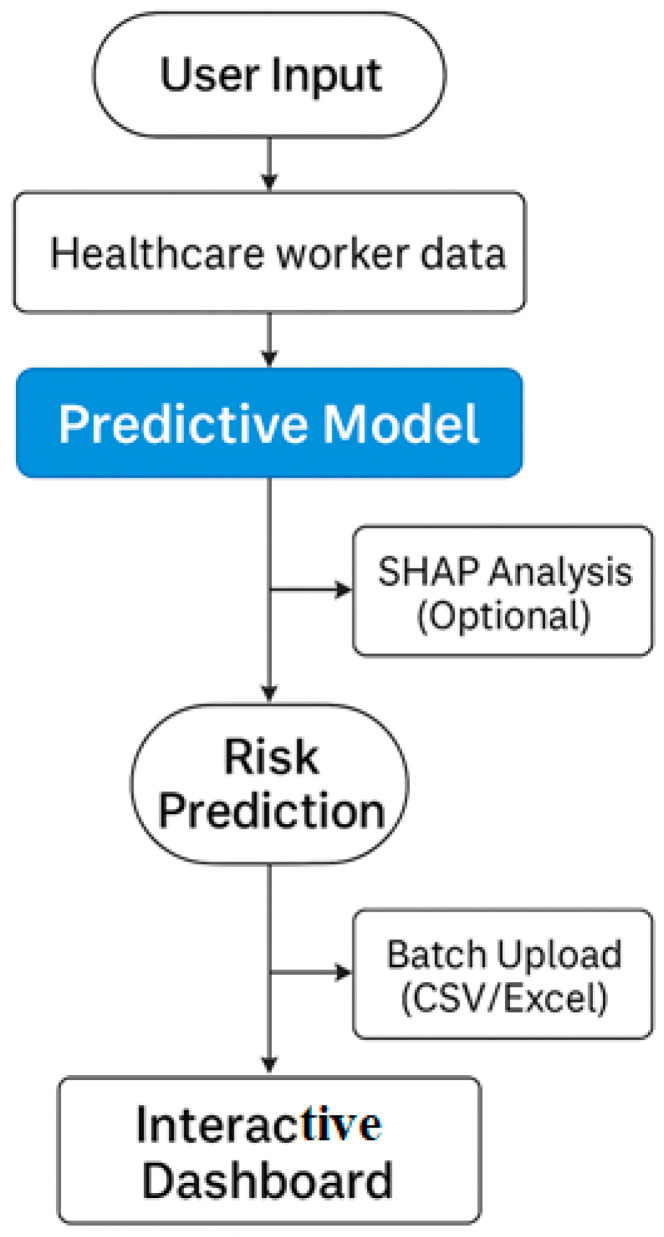
Workflow of the deployed Streamlit application 1.35.0 for healthcare worker risk prediction.

To complement the interface, Figure 31 presents the underlying workflow of the deployed application. This diagram shows the full process from user input (either manual entry or batch upload) through preprocessing, prediction via the trained stacked ensemble models, and delivery of risk estimates and SHAP-based feature explanations. This workflow highlights the transparent, modular design that allows for the rapid adaptation to additional occupational health outcomes or integration into institutional electronic health record systems.

## 4. Discussion

This study evaluated multiple machine-learning pipelines for predicting three key occupational health outcomes—Burnout, Long COVID, and Extended Medical Leave—among hospital staff. By curating a structured dataset of 1244 health records enriched with occupational, demographic, and comorbidity features, we systematically compared the performance of a lightweight transformer model (FTTransformerLite), an enhanced transformer with attention blocks, and a stacked ensemble combining neural and traditional classifiers. This dataset size is substantial for occupational health predictive modeling and exceeds most published AI studies in this niche, offering diverse representation across roles, tenure, and health status.

### 4.1. Model Performance and Comparative Insights

Our findings show that model choice substantially influences predictive performance in occupational health risk prediction. The FTTransformerLite model with Optuna-based tuning served as a robust yet lightweight baseline, but deeper architectures and ensemble strategies consistently delivered higher ROC-AUC and F1 scores.

The Transformer-Lite with attention blocks yielded notable performance gains—particularly for Long COVID and Extended Leave—likely due to its ability to model nonlinear, high-order feature interactions [30,31]. This capability is essential for syndromes like Long COVID, where the pathophysiology and symptom trajectories are multifactorial and heterogeneous [32].

The Stacked Ensemble combining FTTransformerLite, XGBoost, and Logistic Regression achieved the best overall results, with ROC-AUC values > 0.93 and F1 scores approaching 0.90 for Long COVID and Extended Leave. These improvements were statistically significant in bootstrap tests compared to simpler baselines (*p* < 0.0001 for Long COVID and Extended Leave). For Burnout, improvements were smaller but still statistically significant over Logistic Regression (*p* = 0.0355).

These results align with prior evidence that ensembles of diverse learners yield better robustness and generalization in healthcare AI [33,34]. The statistical confirmation of performance gains strengthens the case for real-world deployment, where predictive accuracy directly impacts early intervention, workforce planning, and targeted occupational health measures.

### 4.2. Clinical Interpretability Through Feature Attribution

To facilitate clinical interpretability, we combined two complementary approaches:

1. Permutation Feature Importance using a Random Forest surrogate model for model-agnostic global ranking of predictors.
2. SHapley Additive exPlanations (SHAP) applied to the XGBoost component of the Stacked Ensemble to capture the directionality and magnitude of feature effects at both the global and individual levels.

For the Extended Leave prediction task, permutation importance highlighted the following:

- Cancer History, Pulmonary Conditions, and Obesity—consistent with established predictors of prolonged work absence and medical fragility [35,36];
- Age and Time in Hospital—reflecting cumulative exposure and occupational aging effects [37];
- Spinal and Cardiometabolic conditions—underscoring the biomechanical and systemic burden faced by long-serving clinical staff.

The SHAP analysis (Figure 29) confirmed and extended these findings, showing that Time in Hospital, Age, and Job Role strongly increased the predicted risk across all three outcomes, while condition-specific comorbidities (e.g., Cancer History for Extended Leave) were dominant in individual tasks. The color encoding in SHAP plots illustrates how high or low feature values shift predictions toward a higher or lower risk, providing instance-level transparency critical for clinical adoption.

These findings are clinically coherent and medically explainable, reinforcing the credibility of the models and enabling the potential deployment for targeted occupational health interventions. Furthermore, the use of Focal Loss during training ensured sensitivity to minority classes (i.e., positive cases), improving F1 scores—a crucial consideration in healthcare where false negatives can have significant consequences [38].

### 4.3. Model Deployment and Practical Utility

To translate our predictive models into a usable clinical decision-support tool, we deployed the trained stacked ensemble into a Streamlit-based web application (Figure 29). The interface allows users to carry out the following:

- Enter structured demographic, occupational, and clinical data via intuitive dropdown menus, sliders, and radio buttons;
- Instantly obtain probabilistic predictions for Burnout, Long COVID, and Extended Sick Leave;
- Access explainability mode, which generates SHAP-based feature attribution plots to transparently show how each feature contributes to the predicted risk for an individual;
- Upload batch CSV/Excel files for group-level workforce risk stratification.

The app demonstrated a sub-second latency per prediction in testing and maintained consistent results with those obtained in the held-out validation set. By making advanced AI predictions available through a low-friction, browser-based interface, the tool facilitates the integration into occupational health workflows, enabling the proactive identification of at-risk healthcare staff and supporting early intervention strategies.

### 4.4. Practical and Operational Implications

These models—particularly the stacked ensemble—could be incorporated into institutional occupational health dashboards or early warning systems. Predicting the burnout and long medical leave risk proactively would enable interventions such as modified duties, proactive referrals to mental health support, or ergonomic reassessment.

Furthermore, the high performance on the Long COVID prediction has particular relevance in post-pandemic workforce planning, where chronic symptoms continue to affect the healthcare capacity globally [39,40].

### 4.5. Limitations and Future Directions

Despite promising results, several limitations must be noted:

- Single-site dataset—The dataset was drawn from a single hospital system in Romania, which may limit the generalizability to other healthcare systems, cultural settings, and epidemiological contexts. Differences in the workforce composition, occupational health policies, and delivery structures could influence the model performance elsewhere. External validation using multi-institutional and multinational datasets is essential in order to confirm robustness. Thus, the model generalizability outside of this single institution remains limited and requires independent validation.
- Self-reported outcomes—Burnout and, to some extent, Long COVID status were self-reported, which introduces a potential recall bias and subjective interpretation. Such biases may affect the reliability of the ground truth labels, especially for psychosocial constructs like burnout that are sensitive to context and perception.
- Binary simplification of multidimensional conditions—Both burnout and Long COVID are complex, multidimensional syndromes with heterogeneous manifestations and severities. In this study, they were operationalized as binary outcomes (present/absent) to match the available institutional data. While necessary for modeling feasibility, this is an oversimplification that collapses spectrum conditions into a single label. Future work should incorporate graded burnout inventories and validated Long COVID severity scores to better capture the heterogeneity.
- Algorithmic bias risks—The dataset composition (e.g., a predominance of nurses, ~85% female participants, and an underrepresentation of younger staff) introduces the risk that models may over- or under-estimate risks for certain subgroups. In addition, strong predictors such as obesity and cancer history raise concerns about potential stigmatization or discrimination if predictions were misapplied in HR contexts. To mitigate these risks, we applied class balancing, stratified sampling, and SHAP-based interpretability. We also emphasize that the tool is intended for occupational health support, not punitive decision-making. Nevertheless, fairness analyses across subgroups and explicit de-biasing strategies remain important directions for future work.
- Synthetic oversampling risks—While SMOTE oversampling was effective for the class balance and improved sensitivity to minority outcomes, it may artificially inflate the signal for rare classes and create synthetic patterns that diverge from the real-world variability. This could limit the model reliability in unseen populations if not addressed through external validation. To minimize this risk, we combined SMOTE with stratified cross-validation and robust testing, though we acknowledge that synthetic patterns remain a limitation.
- Data modality limitations—Although structured features were extensively curated, the dataset did not include unstructured data such as free-text clinician notes, psychometric survey responses, or qualitative burnout assessments. Incorporating such multimodal data sources could improve the prediction accuracy for complex outcomes like burnout.
- Future research directions—Future work should explore multi-modal models integrating EHR text, wearable device data, or longitudinal symptom tracking to improve the predictive accuracy and clinical relevance. Additionally, a fairness and bias analysis should be conducted to ensure the equitable deployment across subgroups (e.g., by gender, job role, and tenure) [41].

### 4.6. Contribution to the Field

This work adds to the growing field of explainable AI for workforce health monitoring. It demonstrates that transformer-based models, when properly regularized and combined with ensemble strategies, can achieve both predictive power and clinical transparency—two essential conditions for real-world deployment. It also highlights the importance of domain-specific encoding, careful handling of class imbalance, and robust interpretability to gain trust in occupational health applications [26,42].

A key contribution of this study is the full deployment of predictive models in a real-world application, facilitating clinician access to individualized risk forecasts. By integrating model inference with a user-friendly front-end, we bridged the gap between computational modeling and operational use. Tools like this app can be integrated into occupational health screening workflows and may assist with resource planning, early intervention, and burnout prevention. Furthermore, by training separate, optimized models for each outcome within a single operational platform, this study provides a multi-outcome occupational health decision-support system that reflects the realities of clinical practice.

## 5. Conclusions

This study presents a comprehensive machine-learning framework for predicting occupational health risks—specifically Burnout, Long COVID, and Extended Medical Leave—among healthcare professionals. By leveraging a curated dataset of over 1200 Romanian healthcare workers and more than 20 clinical and occupational features, we developed a series of predictive models grounded in deep learning (FTTransformerLite), transformer-inspired attention mechanisms, and ensemble learning.

Our results demonstrate that transformer-based architectures can effectively model complex tabular healthcare data, especially when combined with advanced loss functions like Focal Loss and optimized using hyperparameter tuning frameworks such as Optuna. Furthermore, stacking FTTransformerLite with traditional models like XGBoost and Logistic Regression consistently improved performance across all outcomes, achieving ROC-AUC values exceeding 0.93 for Extended Leave and Long COVID.

Beyond predictive accuracy, we emphasized model interpretability through the permutation-based feature importance and clinical relevance of the top predictors, including comorbidity burden, age, and occupational exposure. To support real-world applicability, we deployed our final ensemble models into a user-facing application built using the BOLT framework. This interactive tool allows healthcare administrators and clinicians to assess individual or group-level risk profiles using real-time predictions and standardized inputs.

In conclusion, our work provides not only accurate and interpretable models for critical occupational health outcomes but also a practical deployment pipeline to enable integration into clinical and organizational workflows. This translational AI approach holds significant potential for improving occupational health surveillance, workforce planning, and early preventive intervention among healthcare workers.

## Figures and Tables

**Table 1:** Clinical and ordinal encoding strategies for comorbidities—summary of medically informed encoding schemes for categorical predictors based on severity and clinical progression.

Feature	Encoding Strategy
HTA	Ordinal (0–3): Based on ESC/WHO guidelines (Grade 1–3)
Other Cardiovascular Diseases	Ordinal (0–3): Mild dyslipidemia to severe heart failure
Diabetes	Ordinal (0–3): None, prediabetes, type 1, type 2
Spinal Conditions	Ordinal (0–2): Degeneration → Instability → Surgery
Other Musculoskeletal Conditions	Ordinal (0–2): Localized → Systemic/degenerative
Thyroid Conditions	Ordinal (0–2): None → Functional → Structural/Oncologic
Pulmonary Conditions	Ordinal (0–2): None → Asthma/Apnea → COPD/Fibrosis
Obesity	Ordinal (0–2): Normal → Overweight → Obese (Grades I–III)
Cancer History	Binary: 0 = No history, 1 = Cancer history
Smoker	Binary: 0 = Non-smoker, 1 = Smoker

**Table 2:** Binary encoding of target health outcomes—target labels and their respective binary encoding for classification modeling.

Target	Class 0	Class 1
Burnout	“No”, 0	“Yes”, “Burnout”
Long COVID	“No”, 0	“Yes”, “Long COVID symptoms”
Extended Sick Leave	“No”, 0	“Yes”, “Medical Leave”

**Table 3:** Summary of predictor variables: type, encoding scheme, and clinical rationale.

Variable	Type	Encoding	Clinical/Occupational Rationale
**Job Role**	Categorical (Nominal)	Ordinal Grouping (0–5)	Groups reflect exposure risk and work nature (e.g., direct vs. non-clinical care)
**Sex**	Binary	0 = Female, 1 = Male	Epidemiologically relevant in COVID, burnout, and long leave risk
**Age**	Continuous	Raw (in years)	Age influences comorbidities and recovery outcomes
**Time in Hospital**	Continuous	Raw (years of tenure)	Proxy for occupational exposure duration and cumulative strain
**Smoker**	Binary	0 = Non-smoker, 1 = Smoker	Risk factor for pulmonary disease, Long COVID, and cardiovascular burden
**HTA (Hypertension)**	Ordinal	0–3	Based on WHO/ESC classification: from none to grade III hypertension
**Other Cardiovascular Diseases**	Ordinal	0–3	Ranges from mild dyslipidemia to congestive heart failure
**Diabetes**	Ordinal	0 = None, 1 = Prediabetes, 2 = Type 2, 3 = Type 1	Progression captures metabolic risk trajectory
**Spinal Conditions**	Ordinal	0 = None, 1 = Moderate, 2 = Severe/surgical	Reflects biomechanical stress in clinical roles
**Other Musculoskeletal Conditions**	Ordinal	0 = None, 1 = Localized, 2 = Systemic	Includes repetitive strain or widespread degenerative pathology
**Thyroid Conditions**	Ordinal	0 = None, 1 = Functional, 2 = Structural/Severe	Captures impact on energy, mood, and metabolic control
**Pulmonary Conditions**	Ordinal	0 = None, 1 = Mild–Moderate, 2 = Severe	Respiratory impairment linked to work capacity and Long COVID risk
**Obesity**	Ordinal	0 = Normal, 1 = Overweight, 2 = Obese	Obesity severity is predictive of many health outcomes
**Cancer History**	Binary	0 = No, 1 = Yes	History of cancer impacts risk of absenteeism, fatigue, and relapse

**Table 4:** Missingness report across predictive features and targets.

Variable	Missing Values (%)
Job Role	0.0%
Sex	0.0%
Age	0.0%
Time in Hospital	0.0%
Smoker	0.2%
HTA	0.0%
Other Cardiovascular Diseases	0.0%
Diabetes	0.0%
Spinal Conditions	0.0%
Other Musculoskeletal Conditions	0.0%
Thyroid Conditions	0.0%
Pulmonary Conditions	0.0%
Obesity	0.0%
Cancer History	0.0%
Burnout (Target)	0.2%
Long COVID (Target)	0.4%
Extended Sick Leave (Target)	0.2%

**Table 5:** Hyperparameter search space and optimization ranges used in the Optuna study for tuning the FTTransformerLite model. The study explored categorical, uniform, and log-uniform parameter distributions over 15 trials, with F1 score as the optimization objective.

Parameter	Range
Hidden dimension	[32, 64, 128] (categorical)
Dropout rate	Uniform [0.1, 0.5]
Learning rate	Log-uniform [1 × 10^−4^, 5 × 10^−3^]

**Table 6:** Classification performance of predictive models for each target outcome.

Outcome	Model	Accuracy	ROC-AUC	F1 Score
Burnout	FTTransformerLite (Optuna)	0.5868	0.6331	0.6124
	Transformer-Lite + Attention	0.6354	0.6974	0.6512
	Stacked Ensemble	**0.6354**	**0.6974**	**0.6512**
Long COVID	FTTransformerLite (Optuna)	0.6334	0.6738	0.6200
	Transformer-Lite + Attention	0.8724	0.9259	0.8635
	Stacked Ensemble	**0.8973**	**0.9336**	**0.8941**
Ext. Leave	FTTransformerLite (Optuna)	0.5663	0.6223	0.5424
	Transformer-Lite + Attention	0.8724	0.9259	0.8635
	Stacked Ensemble	**0.8973**	**0.9336**	**0.8941**

Note: Best-performing models are bolded and marked with bold font.

**Table 7:** Bootstrapped pairwise comparisons of ROC-AUC performance between the Stacked Ensemble model and two baseline classifiers (Logistic Regression, and Random Forest) across three occupational health outcomes (Burnout, Long COVID, and Extended Leave). Results are reported as the mean difference in AUC (ΔAUC), 95% confidence intervals (CI), and empirical *p*-values from 10,000 bootstrap resamples. Positive ΔAUC values indicate higher performance for the Stacked Ensemble.

Outcome	Comparison	Mean ΔAUC	95% CI	*p*-Value
Burnout	Ensemble vs. Logistic Regression	0.0506	[−0.0030, 0.1065]	0.0355
Burnout	Ensemble vs. Random Forest	0.0143	[−0.0333, 0.0602]	0.2745
Long COVID	Ensemble vs. Logistic Regression	0.2955	[0.2426, 0.3481]	**<0.0001**
Long COVID	Ensemble vs. Random Forest	−0.0040	[−0.0198, 0.0121]	0.3225
Extended Leave	Ensemble vs. Logistic Regression	0.2083	[0.1645, 0.2532]	**<0.0001**
Extended Leave	Ensemble vs. Random Forest	−0.0262	[−0.0428, −0.0104]	**<0.0001**

Note: Bold values indicate significance at *p* < 0.05. *p*-values less than 0.0001 are reported as *p* < 0.0001.

**Table 8:** Top 10 features for Extended Leave (Random Forest permutation importance).

Rank	Feature	Importance Score
1	Cancer History	0.137
2	Pulmonary Conditions	0.102
3	Obesity	0.098
4	Age	0.081
5	Spinal Conditions	0.079
6	Time in Hospital	0.072
7	HTA (Hypertension)	0.065
8	Other Cardiovascular Diseases	0.057
9	Diabetes	0.049
10	Thyroid Conditions	0.042

## Data Availability

The dataset analyzed in this study is stored securely on Google Drive and is not publicly available due to privacy restrictions and the sensitive nature of personal health information. Access can be granted upon reasonable request to the corresponding author e-mail (irina-luciana.gurzu@umfiasi.ro) and subject to approval by the hospital’s ethics committee.

## Supplementary Materials

The following supporting information can be downloaded at: https://www.mdpi.com/article/10.3390/healthcare13182266/s1, Table S1: Performance of baseline logistic regression models for predicting burnout, Long COVID, and extended medical leave among healthcare workers, using the curated feature set without SMOTE oversampling or feature engineering. Models were trained with standardized predictors and evaluated on a held-out stratified test set. Metrics are reported as Accuracy, Area Under the Receiver Operating Characteristic Curve (ROC-AUC), and F1 Score.
