# Supplementary material for: An Integrated AI Framework for Occupational Health: Predicting Burnout, Long COVID, and Extended Sick Leave in Healthcare Workers
Source: Healthcare (Basel). 2025 Sep 10;13(18):2266. doi: 10.3390/healthcare13182266 (PMC12469670; doi:10.3390/healthcare13182266)
Supplement: Supplementary file 1 [file healthcare-13-02266-s001.zip › healthcare-3840827-supplementary.pdf]

Supplementary Table

We add this as **Supplementary Table S1** in this supplementary section, clearly showing the performance of a **vanilla Logistic Regression**baseline. We run logistic regression **without** SMOTE, without feature engineering, and without advanced architectures, just raw numeric and ordinal-encoded predictors → trained on the same split for fairness.

Supplementary Table S1. Performance of baseline logistic regression models for predicting burnout, Long COVID, and extended medical leave among healthcare workers, using the curated feature set without SMOTE oversampling or feature engineering. Models were trained with standardized predictors and evaluated on a held-out stratified test set. Metrics are reported as Accuracy, Area Under the Receiver Operating Characteristic Curve (ROC-AUC), and F1 Score.

**Outcome**	**Model**	**Accuracy**	**ROC-AUC**	**F1 Score**
Burnout	Logistic Regression (raw)	0.58	0.62	0.41
Long COVID	Logistic Regression (raw)	0.87	0.61	0.00
Extended Leave	Logistic Regression (raw)	0.88	0.65	0.25
